# Nanoparticle-assisted targeting of heart lesions with cardiac myofibroblasts: Combined gene and cell therapy

**DOI:** 10.7150/thno.103816

**Published:** 2025-03-18

**Authors:** Miriam Schiffer, Kevin Wagner, Esther Carls, Julia Nicke, Michael Hesse, Raluca M. Fratila, Staffan Hildebrand, Dietmar Eberbeck, Timo Mohr, Mona Malek Mohammadi, Jesus Martinez de la Fuente, Bernd K. Fleischmann, Wilhelm Roell

**Affiliations:** 1 Institute of Physiology I, Medical Faculty, University of Bonn, Venusberg-Campus 1, 53127 Bonn, Germany.; 2 Department of Cardiac Surgery, University Hospital Bonn, Venusberg-Campus 1, 53127 Bonn, Germany.; 3 Instituto de Nanociencia y Materiales de Aragón, INMA (CSIC-Universidad de Zaragoza), Campus Río Ebro, Edificio CIRCE, despacho 00.070 C/ Mariano Esquillor, s/n - 50018 Zaragoza, Spain.; 4 Centro de Investigación Biomédica en Red de Bioingeniería, Biomateriales y Nanomedicina (CIBER-BBN), Av. Monforte de Lemos, 3-5. Pabellón 11. Planta 0 28029 Madrid, Spain.; 5 Institute of Pharmacology and Toxicology, Medical Faculty, University of Bonn, Biomedizinisches Zentrum, Venusberg-Campus 1, 53127 Bonn, Germany; 6 Physikalisch-Technische Bundesanstalt (PTB), Abbestraße 2-12, 10587 Berlin, Germany.

**Keywords:** myocardial infarction, cardiac myofibroblasts, gene and cell therapy, ventricular arrhythmias, Connexin 43

## Abstract

**Rationale:** The cardiac scar is an area rich in collagen. It is populated by myofibroblasts and lacks Connexin 43 expressing cardiomyocytes. Myocardial infarctions have so far proven little amenable to gene- and cell-based therapeutic interventions. Our aim was to establish an experimental approach with translational potential for effective cell-based gene therapy of the cardiac scar.

**Methods:** We have developed a targeting strategy for myocardial infarctions by grafting *ex vivo* lentivirus-transduced and magnetic nanoparticle-loaded embryonic cardiac myofibroblasts into mouse hearts with magnetic steering.

**Results:** Our approach yielded highly efficient targeting and cell grafting into the cardiac scar. Engraftment rates of myofibroblasts proved very high (30% of injected cells) due to cell proliferation and a low apoptosis rate. We also demonstrate that grafting lentivirus-transduced Connexin 43 overexpressing myofibroblasts into the lesion resulted in increased Connexin 43 protein content and strong protection against ventricular arrhythmias *in vivo*, as their incidence was reduced by ~ 50% at 2- and 8 weeks after myocardial infarction.

**Conclusion:** The combination of *ex vivo* gene and *in vivo* cell therapy, along with magnetic steering of cardiac myofibroblasts, enables, efficient targeting of the cardiac scar and can even modulate its functional properties.

## Introduction

Cardiovascular diseases, particularly myocardial infarctions (MI) are among the leading causes of death worldwide. Common and potentially life-threatening complications during both, the acute and chronic phases of severe ischemic heart disease include ventricular tachycardias (VT), primarily triggered by re-entry mechanisms 1, 2. In addition to optimal medical treatment 3, 4, interventional electrophysiology 5, and the implantation of cardioverter defibrillators (ICDs, 6), there is currently no causal therapy available to treat and improve the long-term outcome of VT. One reason is that the myocardial scar has so far proven to be particularly challenging for gene therapy-based targeting approaches. In fact, the most widely used viral vectors in both rodents and humans, such as adeno-associated viruses (AAV) and lentivirus (LV), have a low efficiency in transducing cardiac (myo)fibroblasts (FB) in the scar *in vivo* 7. Similarly, cell therapy is challenging, as the number of grafted embryonic cardiomyocytes (eCM) strongly declines over time following injections into the cardiac lesion 8-10. We have shown that this is due to the retrograde flux of eCM out of the injection channel and to eCM apoptosis, most likely because of the adverse local conditions. We have successfully improved engraftment rates of eCM by loading the cells with magnetic nanoparticles (MNP) and magnet-assisted injection into the lesion 10, 11.

Upon MI, quiescent cardiac FB become activated and form and stabilize the cardiac scar 12. These cells are also present in the non-infarcted mammalian heart, where they comprise approximately 20-27% of the total cell content 13. One advantage is that cardiac FB can be easily harvested and expanded *in vitro* and that these cells are more resistant to adverse local conditions 14-16. Given the poor results with direct viral targeting of MI and our success with cardiac cell grafting, we explored the utility of a combined *ex vivo* gene and *in vivo* cell therapy approach. We combined *ex vivo* MNP loading of embryonic cardiac activated myofibroblasts (mFB) with magnetic steering for intramyocardial cell injections, which resulted in strongly enhanced engraftment rates of these activated mFB. Next, we probed the functional impact of this strategy by overexpressing the gap junction protein Connexin43 (Cx43) in mFB *in vitro*, followed by MNP loading and intracardiac injections in infarcted mouse hearts. This yielded a strong anti-VT effect *in vivo* at 2- and 8 weeks post-CI underscoring the utility and impact of this approach. Thus, *ex vivo* gene therapy of mFB in combination with magnet-guided grafting is a promising strategy for efficiently targeting and modulating the functional properties of the cardiac scar.

## Materials and methods

### Preparation of the PMAO-MNP

Briefly (for details see 17, 18), the reagents used for MNP synthesis were purchased from Sigma-Aldrich (Taufkirchen, Germany) and Merck (Darmstadt, Germany). Monodisperse hydrophobic iron oxide MNP (diameter = 12 nm) were obtained following a seed-mediated growth method based on the thermal decomposition of Fe(III) acetylacetonate.

#### Synthesis of 6 nm iron oxide nanoparticle seeds

In a three-neck flask equipped with mechanical stirring, a temperature probe and a water condenser, Fe(acac)3 (0.71 g, 2.01 mmol), 1,2-hexadecanediol (2.58 g, 9.98 mmol), oleic acid (2 ml, 5.65 mmol) and oleylamine (2 ml, 4.22 mmol) were added, followed by 40 ml of benzyl ether. The reaction mixture was degassed, nitrogen flushed 3 times, heated to 200°C (heating rate: 3°C/min), and maintained at this temperature for 2 h to promote nucleation. The temperature was increased to 305°C (heating rate: 10°C/min), maintained for 2 h at 305°C and then the reaction mixture cooled down to room temperature (RT). The black nanoparticle suspension was washed 3 times with ethanol (to precipitate the MNP) and hexane (to redisperse the MNP), and the MNP were redispersed in a mixture of hexane, oleic acid and oleylamine (ratio 90:5:5), and stored at 4 ºC until further use.

#### Growth of 12 nm iron oxide nanoparticles

To obtain 12 nm nanoparticles, 40 mg of the 6 nm MNP seeds in hexane were added to a mixture containing Fe(acac)3 (1.42 g, 4.02 mmol), 1,2- hexadecanediol (5.16 g, 19.97 mmol), oleic acid (1 ml, 3.12 mmol), oleylamine (1 ml, 3.06 mmol), and benzyl ether (40 ml). The mixture was heated to 100°C (3°C/min) and maintained for 30 min to remove the hexane, then heated to 200°C (3°C /min) and maintained at this temperature for 1 h. Finally, the temperature was further increased to 305°C (10°C/min) and maintained for 1 h. The reaction mixture was cooled down to RT, and the MNP were washed and stored as indicated before.

Water transfer of the MNP: 140 mg of polymaleic anhydride-alt-1-octadecene (PMAO, MW 30000-50000 Da) were added to a 500 ml round-bottomed flask containing 15 ml of chloroform. The polymer was dissolved under magnetic stirring and 2 mg (3.77 μmol) of 5-carboxytetramethylrhodamine (TAMRA) cadaverine dissolved in 3 ml of ethanol were added. The reaction mixture was stirred overnight at RT, protected from light. The next day, 80 ml of chloroform was added (to a final total volume of 98 ml) and the flask was placed in an ultrasonic bath. Then, 10 mg of the hydrophobic 12 nm MNP (previously washed 3 times with ethanol and resuspended in 2 ml of chloroform) were added drop-wise, and the mixture was kept in the bath for 15 min at RT. Chloroform was then evaporated using a rotary evaporator until a final volume of 5-10 ml, 10 ml of Milli-Q water and 10 ml of 0.1 M NaOH were added simultaneously (foaming occurs due to the hydrolysis of the maleic anhydride groups of PMAO) and the solvent was removed at 70°C increasing the vacuum progressively until the complete disappearance of foam. The solution was then filtered using a 0.2 μm syringe filter. Finally, unbound PMAO was eliminated through four ultracentrifugation steps of 2 hours at 24000 rpm at 14 ºC. All MNP suspensions were sterilised by filtration using 0.22 μm MilliPore® filters before addition to cell cultures.

### Characterisation of the MNP

#### Transmission Electron Microscopy (TEM)

TEM analysis was performed using a FEI Tecnai T20 instrument at an accelerating voltage between 80 - 200 kV (Laboratorio de Microscopias Avanzadas LMA, University of Zaragoza). TEM samples were prepared by drop casting the MNP suspension on carbon-coated copper grids (Electron Microscopy Sciences, EMS) and dried under ambient conditions. Particle size and size distribution were determined using the ImageJ software measuring the diameter of at least 200 MNP per image (Figure S1A).

#### Dynamic light scattering (DLS) and ζ -potential measurements

A Malvern Instruments Zetasizer NANO was used for the determination of the hydrodynamic diameter (by DLS) and for the measurement of the zeta potential of the MNP. All samples were irradiated with a 633 nm He-Ne laser. Samples were prepared in Milli-Q water (0.05 mg/ml), filtered and the pH was adjusted to 7. Each sample was measured 5 times combining 10 runs per measurement at 25°C. Results were treated using the Malvern software Zetasizer Nano 7.03 (Figure S1B).

### Lentivirus (LV) production and titration

The preparation, purification, and titration of self-inactivating LV-vectors (rrl-CMV-IRES-eGFP or rrl-CMV-Cx43-IRES-eGFP) were performed, as described in detail earlier 7, 19. The eGFP expression differs between the two lentiviral constructs, because of the bicistronic LV-Cx43-eGFP construct (Figure 2A, Figure S2E) enabling the co-expression of Cx43 and the reporter eGFP under the control of the same (CMV) promoter to monitor transduced cells expressing exogenous Cx43 20.

### LDH and MTT toxicity assays

NIH-3T3 fibroblasts (3T3-FB, 1.0 x 10^4^ cells, in 24-well plates) were cultured in 10% FCS/DMEM (Gibco, Life Technologies, Life Technologies, Carlsbad, CA, US). 3T3-FB were incubated with MNP (SoMag5-MNP 10 or PMAO-MNP were added to cell culture medium) in concentrations ranging from 5 pg - 100 pg Fe/cell for 60 min (24 well plate, Chemicell, Berlin, Germany). The toxicity of MNP was determined 24 h upon MNP treatment using the LDH- toxicity Assay (Promega Corp, Fitchburgh, WI, US) according to the manufacturer's instructions. Results are given in toxicity levels [%] per pg Fe/cell. Proliferation rate of 3T3-FB was determined 24 h after MNP treatment via MultiTox-Fluor Multiplex Cytotoxicity Assay (MTT, Promega). As controls, FB were either untreated or treated with 2% DMSO.

### Magnetic Particle Spectrometry (MPS)

For MPS 4.0 x 10^4^ 3T3-FB/well were seeded onto 24-well plates and cultured in 10% FCS/ DMEM (Gibco). Cells were treated for 60 min (at 37°C and 50 rpm on a rotary shaker) with MNP (added to cell culture medium) in different concentrations (15, 25, 50 pg Fe/cell). Afterwards, cells were detached via a 5 min exposure to Trypsin (Gibco), cell numbers were determined and cells were transferred into a particulate-free reaction tube containing 2.5% agarose gel. MPS analysis was done in cooperation with PTB Berlin, as reported before 10, 21, 22.

### Analysis of retained cell number post magnet application *in vitro*

In order to determine the retention of MNP-treated cells, 3T3-FB were seeded with a density of 2.5 x 10^5^ cells/well onto a 6-well plate. After 24 h, cells were treated with MNP by using either 25 or 100 pg Fe/cell iron concentration. After additional 24 h, cells were detached with Trypsin (5 min at 37°C) and the total cell number counted. Thereafter, cells were transferred onto a magnet rack (customised, laterally mounted magnet) and numbers of retained cells were determined, as described before 21, 23.

### Isolation, culturing, LV transduction and MNP loading of murine embryonic cardiac (mFB)

**Scheme 1:**
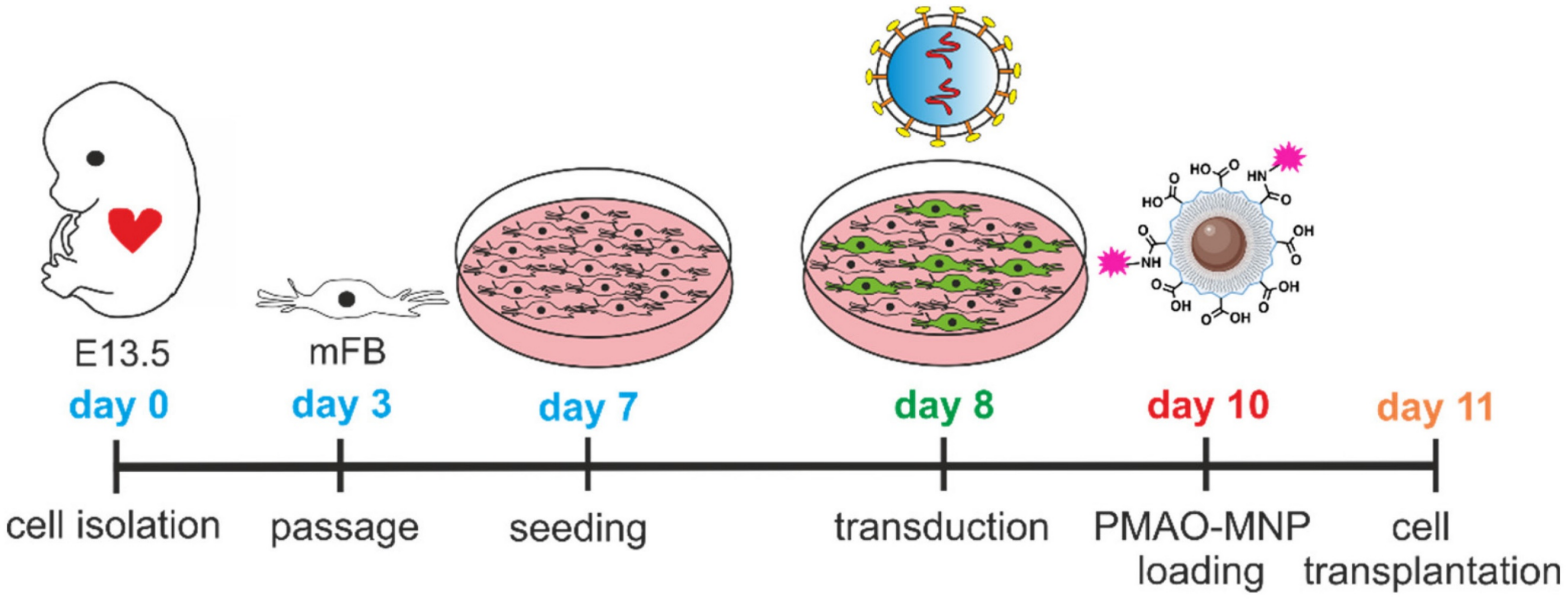
Diagram illustrating the timeline of mFB isolation, culturing, LV transduction, and PMAO-MNP loading prior to grafting. Abbreviations: lentivirus (LV); magnetic nanoparticles (MNP); myofibroblasts (mFB).

For the enrichment of mFB and FB we used published protocols of adhesion plating 24-26. Briefly, hearts of mouse embryos (E13.5, CD1 wild type) were harvested and enzymatically digested for 45 min at 37°C with collagenase type II (520 U/ml, Worthington, NJ US). The isolated cells were plated (0.1% gelatine-coated T75 flasks, approx. 1.0 x 10^7^ cells) and cultured in 10% FCS-containing DMEM (Gibco). Medium was changed every 3 days. At confluence, cells were detached using Accutase (~ 5 min at RT, Merck) and further passaged (one passage at day 3, 1:2 splitting, Scheme 1). To obtain sufficient cell numbers for a series of transplantation experiments, the cultivation time of the cells was at least 11 days, at this time, the cell population was CM-free.

For transplantation experiments, mFB were seeded onto 6-well plates coated with 0.1% gelatine (1.0 x 10^6^ cells/well) and transduced overnight (at day 8) with the LV-constructs (Multiplicity of infection (MOI) = 5). The day after, the medium was changed. For analysing transduction efficiency, 7.5 x 10^3^ mFB were seeded on 24-well plates (coated with 0.1% gelatine) and transduced as described above. After 3 or 17 days following LV transduction, the cells were fixed and analysed by immunohistochemistry.

24 h prior to intramyocardial injections, PMAO-MNP were added to the cell culture medium overnight at 37°C at a concentration of 25 pg Fe/cell (at day 10) without magnetic field application. Prior to surgery, MNP-loaded mFB were detached with Accutase (10 min, RT) and washed at least 3 times with PBS, with 5 min of centrifugation at 1000 rpm each time. Finally, for preparation of the injection solution, mFB were resuspended in 10% DMEM at a concentration of 0.4x10^5^ cells/µl. Then, 5 µl of this solution containing a total amount of 2.0 x 10^5^ mFB were injected intramyocardially into each animal (at day 11).

### Isolation of transgenic murine neonatal cardiac myofibroblasts (exoFB) for transplantation

Transgenic exoFB for grafting experiments were obtained from culturing cardiac FB of pups (P3) of the double-transgenic murine Cre-reporter line: mTmG (Gt(ROSA)^26Sortm4(ACTB-tdTomato,-EGFP)Luo^) 27 x Tcf21^MCM^ 28. For some experiments, Tcf21-dependent eGFP expression was induced by treating neonates with tamoxifen (Sigma, intragastric injection, 0.03 mg/g body weight) daily from P0 to P2 (3 doses in total). As the recombination rate was, as reported earlier 29, only in the range of 60-80% and as approximately 85% of tomato^+^ cell fraction were mFB (Figure S4A), for the majority of experiments, tamoxifen was not applied. Neonatal hearts were harvested at P3, and exoFB isolated using collagenase type II. exoFB were cultivated in 10% FCS containing DMEM for 3 days and then passaged and seeded onto 0.1% gelatine coated 6-well plates. On day 4 of cultivation, exoFB were treated overnight with MNP in cell culture medium (PMAO-MNP, 25 pg Fe/cell). On day 5, exoFB were detached and prepared for grafting in cryoinjured CD1 wild-type mice (as described above). Engraftment rates of exoFB were quantified by counting eGFP^+^/tomato^+^ cells (after tamoxifen treatment, n = 3) or tomato^+^ cells (no tamoxifen treatment, n = 9) in heart sections (Figure 4F). For the characterisation and quantitation of exoFB enrichment, isolated cardiac cells from transgenic hearts (4 with tamoxifen treatment (Figure S4A) and 10 with no tamoxifen treatment (Figure 4B)) were immunostained against different cell markers after 5 and 12 days in cell culture, respectively.

### Transplantation of mFB or exoFB into infarcted mouse hearts

**Scheme 2:**
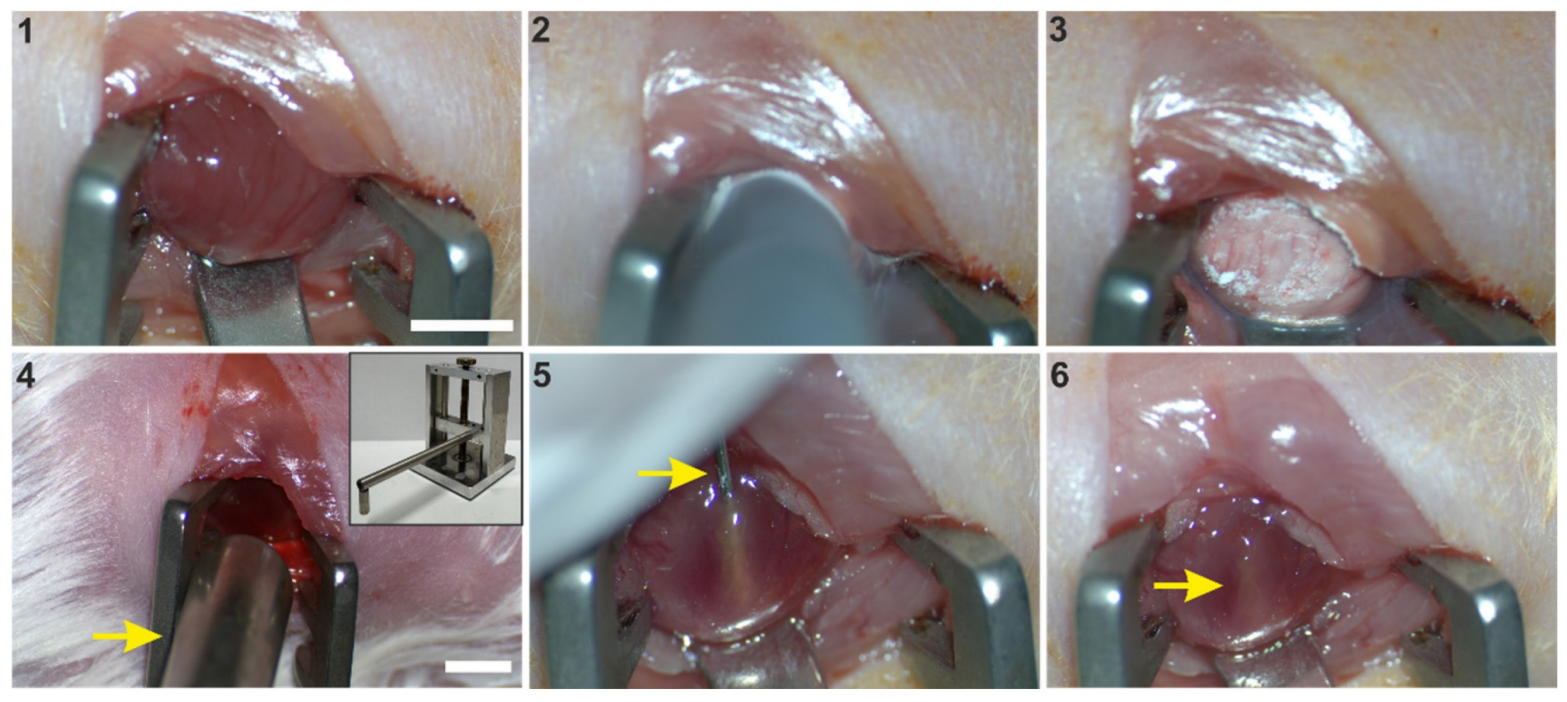
CI and magnet-assisted injection of mFB. 1: Left sided thoracotomy and exposition of the free left ventricular wall, 2: CI by use of a precooled copper probe (diameter of 3.5 mm), 3: Frozen myocardium, 4: Positioning of the 1.3 T rod magnet (arrow) opposite to the freshly infarcted heart surface, 5: Injection of mFB via a 29 G needle (arrow), inserted into the central part of the lesion, 6: Pocket of injected cells (arrow) after removal of the injection needle. Bar: 5 mm. Abbreviations: cryoinjury (CI); myofibroblasts (mFB).

Mice received buprenorphine (0.1 mg/kg s.c.; Burprenovet®, Elanco GmbH, Cuxhaven, Germany) as an analgesic 30 min prior to surgery and up to 3 days post-CI 3 times per day. Eye ointment (Bepanthen ® Augen- und Nasensalbe, Bayer AG, Leverkusen, Germany) was applied during the operation on the warming plate. Under inhalation anesthesia (Isoflurane 1.5- 2.0 Vol.%, IsoFlo®, Zoetis GmbH, Munich, Germany) and sterile conditions a left anterolateral thoracotomy was performed in the 5^th^ intercostal space in adult (10-12 weeks) CD1 wildtype female mice. The heart was elevated using a small spatula (Scheme 2, picture 1), and a round copper probe (pre-cooled in liquid nitrogen, 3.5 mm in diameter) was placed on the left anterolateral ventricular wall three times for 15 seconds with gentle pressure (Scheme 2, picture 2,3). A bar magnet (1.3 T, diameter 5 mm) was then installed at a distance of 5 mm above the lesion area using a micromanipulator (Scheme 2, Picture 4). The magnetic field was applied during the injection procedure and for 10 min afterward, as described before 10. Next, 5µl of the cell suspension (10% DMEM, 0.4x10^5^ cells/µl), containing 2.0 x 10^5^ LV-transduced and MNP-loaded mFB or exoFB, were injected slowly (5 s) into the centre of the lesion using a 10 µl Hamilton syringe equipped with a 29-G needle (Scheme 2, picture 5,6).

Then, the chest was closed, de-aired, and the mice allowed to wake up. Mice received Prednisolon (1 mg/kg diluted in 5 % glucose i.p., Merck) up to 10 days post-CI to reduce the potential immune response to the LV-vectors. Before being returned to the animal house, the mice were observed for several hours under a heat lamp in the laboratory. In the animal house, where the lighting simulates a 12-hour day/night rhythm, the mice were kept in small groups (up to 5 animals) in cages fitted with filtertops and equipped with material for nest building. In addition to the daily checks by the animal keepers, the mice were monitored up to 3 times a day during the first 3 days after the surgery and after that once a day by the lab staff. The state of health of each animal was documented using individual score sheets.

### Functional analysis with echocardiography and electrophysiology *in vivo*

We have chosen the cryoinjury (CI) model because these cardiac lesions are transmural and highly reproducible in size, which is critical for the functional assays.

Transthoracic echocardiography was performed 2- and 8 weeks post-CI, one day before *in vivo* electrophysiology testing. Left ventricular function was measured under inhalative anaesthesia using a Phillips CX50 ultrasound system (ATL-Phillips, Oceanside, CA, USA) equipped with a 15-MHz transducer in short-axis M-mode recordings at the level of the papillary muscles.

*In vivo* electrophysiology testing was performed 2- and 8 weeks post-CI under inhalative anaesthesia (day 14 and 56), as reported earlier 7, 11. Briefly, the tip of a 2 French octapolar mouse-electrophysiological catheter (CIBER Mouse Electrophysiology Catheter, NuMed Inc., Hopkinton, NY, US*)* was inserted into the apex of the right ventricle via right jugular vein while a surface 4-lead ECG was recorded (PowerLab 16/30, LabChart 7, AD Instruments, Pty LTD, Australia). Additionally, bipolar intracardiac electrograms were recorded on atrial, his-bundle and ventricular levels. Electrophysiological vulnerability was tested in extra- and burst stimulus testing. Rectangular stimulation pulses were applied via the apical pair of electrodes by use of a multiprogrammable stimulator (Model 2100, A-M Systems, USA; Stimulus 3.4 software, Institute for Physiology I, University of Bonn, Germany). Extra-stimulus stimulation was performed with up to 3 additional beats (with stepwise S1-S2 or S2-S3 reduction by 5 ms, starting with 90 ms below S1-S1) at S1-S1 cycle lengths of 120 ms, 110 ms, and 100 ms. During burst stimulation, S1-S1 stimulations with cycle lengths starting at 50 ms were performed for 1 s and repeated three times, decreasing by 10 ms until 10 ms. Between stimulation events, the hearts were allowed to recover for at least 4 s. According to the clinical definition and previous publications, a VT was characterised by at least four consecutive ventricular extra beats with atrioventricular dissociation 30.

### Immunofluorescence stainings

For *in vitro* immunostainings, cells were seeded on glass slides (1.0x10^4^ cells/well) in 24-well plates coated with 0.1% gelatine and fixed with 4% formaldehyde (FA, Sigma-Aldrich) for 20 min at RT. The different antibodies were added at concentrations indicated below and diluted in a volume of 80 µl per glass slide. Macroscopic brightfield and fluorescence images of harvested hearts were acquired with a Zeiss Axio Zoom V16 macroscope (Carl Zeiss Microscopy GmbH, Jena, Germany). Hearts were fixed overnight at 4°C with 4% FA and incubated for further 24 h with 20% sucrose solution, before they were embedded in Tissue-Tek® medium (Sakura Finetek, Europe B.V., Umkirch, Germany) and frozen on dry ice. From the frozen hearts, 7 µm thick sections were prepared using a cryotome (Leica Biosystems, Wetzlar, Germany). To detect the transplanted mFB and to analyse Cx43 expression in the scar area, the sections were permeabilised with 0.2% Triton-X-PBS (Sigma- Aldrich) and simultaneously blocked with 5% donkey serum (DS) (Jackson ImmunoResearch, Ely, UK). Then, antibodies (Cx43: 1:800 custom-produced rabbit polyclonal; αSMA: 1:800 mouse monoclonal; Vimentin: 1:1000 chicken polyclonal; CD45: 1:800 rat monoclonal (all Merck); CD31: 1:400 rat monoclonal (Becton Dickinson GmbH, Heidelberg, Germany); Ki67: 1:200 rabbit monoclonal; cTroponinT: 1:200 mouse monoclonal (both Thermo Fisher Scientific GmbH, Dreieich, Germany); PDGFRα: 1:200 goat polyclonal, R&D Systems; Thermo Fisher; Tcf21: 1:200 rabbit, Novus Biologicals, Toronto, Canada; clCaspase3: 1:200 rabbit, Cell Signalling Technology, Cambridge, UK; αActinin: 1:400 mouse monoclonal (Sigma-Aldrich)) were diluted in 5% DS/PBS and applied for 2 h at RT to the tissue sections, followed by 3 washing steps using PBS. Secondary antibodies (all 1:400, host: donkey, Jackson ImmunoResearch) were diluted in Dapi (Thermo Fisher) and applied for 1 h at RT. After washing, slides were covered with aqueous mounting medium (Aqua Polymount, Thermo Fisher) and imaged using confocal (Eclipse Ti, Nikon Europe B.V., Amstelveen, Netherlands) or fluorescence microscopy (BZ-X810, Keyence, Neu-Isenburg, Germany).

### Analysis of infarct size and quantification of collagen content within native and infarcted myocardium

Infarct surface was determined using macroscopic images of the infarcted hearts acquired with an Axio Zoom V16 (Zeiss, 8x magnification). The infarct borders were clearly visible due to the CI model, and the surface area (given in mm^2^) was measured using ImageJ software (NIH, Bethesda, MD, US). For morphometric analysis of infarct volumes, hearts were cryopreserved and sectioned as described above. Over the entire heart, every 300 µm a section was analysed, and the measured infarct area was extrapolated to the total volume of the myocardium. To clearly mark the scar area or to detect changes in collagen content in the native myocardium, heart sections were stained with Sirius red (Sigma-Aldrich) and Fast Green (Fisher-Scientific). Overview images (32x magnification) were taken with a macroscope (Zeiss, Axio Zoom V16) and analysed with ImageJ.

### Determination of wall thickness and cellularity of infarcted myocardium

Cellularity and wall thickness following CI were determined by Dapi staining (Thermo Fisher) of 2 representative slides per heart 2- and 8 weeks post-CI (each n = 3). Nuclei were counted in the border zone and the centre of the lesion in 2 squares (area approximately 0.02 mm^2^/square) per slide and location. The mean nuclei amount of both squares was extrapolated to an area of 0.5 mm^2^. Wall thickness was measured with Image J in the centre of the lesion (given in µm).

### Quantification of engrafted fibroblasts

For quantification of engrafted mFB, hearts were cryopreserved and serially cut (7 µm thickness, as described above). To better visualise the eGFP fluorescence of transduced embryonic mFB prior to cell counting, slides were incubated with anti-eGFP (1:200 goat polyclonal, Santa Cruz Biotechnology, Delaware, US) after antigen retrieval. In addition, slides were incubated with Vimentin (1:1000 chicken polyclonal, Merck) as fibroblast marker (see staining protocol above) and nuclei with Dapi (Thermo Fisher). Images were recorded using an Axio Observer microscope equipped with epifluorescence (Zeiss). The number of engrafted eGFP^+^ or tomato^+^ and Vimentin^+^ mFB was determined in 3 hearts per experimental group. For this purpose, the labelled mFB were counted in a total of 8 slices at a distance of 280 µm per infarct area, including the border zone, and extrapolated to the total volume of the myocardial lesion.

### Fluorescent Recovery After Photobleaching (FRAP)

For FRAP experiments mFB were isolated, seeded (2.0 x 10^5^ cells/well, 6 well plates equipped with glass plates, 0.1% gelatine-coated) and LV transduced as described above. Six days post-transduction mFB were incubated with Calcein AM Violet (0.38 µM, Invitrogen, Thermo Fisher Scientific) diluted in 10% DMEM for 20 min at 37°C. Fluorescence intensity of a cluster of Calcein AM^+^ and eGFP^+^ mFB was measured with a confocal microscope (Nikon Eclipse Ti, 40x objective; CFI Apo Lambda S LWD 40XWI, NA 1.25, Nikon), before a single mFB was bleached with a 410 nm and 488 nm laser pulse (20 s, 2.5 mW). Subsequently, fluorescence images were taken every 10 s over 4 min, and fluorescence intensity was measured. Fluorescence intensities were converted into values between 1 (fluorescence intensity before bleaching) and 0 (fluorescence intensity immediately after bleaching).

### RNA isolation, quantification and RNASeq analysis of samples

RNA from mFB (see above) was isolated (according to manufacturer instruction) by using the RNeasy© Mini Kit (Qiagen, Hilden, Germany). After RNA quantification by absorption measurement, RNA quality was determined with a Bioanalyzer (Agilent Technologies, Waldbronn, Germany) using the RNA 6000 Nano Kit (Agilent). For RNASeq and qPCR analysis, RNA integrity numbers of analysed samples ranged between 9 - 10. A RNA library was generated according to manufacturer instruction by use of the Trio RNASeq library Kit (NuGEN Technologies Inc, San Carlos, CA, US). RNA bulk sequencing was done (EMBL, Heidelberg, Germany) by paired-end sequencing including 10 - 15 million reads.

### Gene expression analysis using TaqMan™ qPCR

The gene expression in transduced mFB was investigated by qPCR using TaqMan assays (Thermo Fisher). For this, 250 ng RNA was transcribed into cDNA using the SuperScriptVILO Kit (Thermo Fisher). Then, 2 µl cDNA was amplified with TaqMan™ qPCR (38 cycles, 3 technical replicates of 3 biological replicates) using master mix (Thermo Fisher) and the following TaqMan assays: eGFP (forward: GAGCGCACCATCTTCTTCAAG, reverse: TGTCGCCCTCGAACTTCAC), Gata4 (#Mm01310448_m1), Tnnt2 (#Mm00441920_m1), Atp2a2 (#Mm01201431_m1) and Caspase1 (#Mm00438023_m1) (all assays: Thermo Fisher, FAM). ΔCT values were calculated with mean sample CT normalized to mean CT values of the endogenous housekeeping gene HPRT (Thermo Fisher, #Mm03024075_m1, VIC) detected via Multiplex qPCR.

### Western blotting

Protein isolation of *in vitro* samples was performed using RIPA buffer (50 mM Tris-HCL pH 7.5, 1% NP-40, 0.25% Na-Desoxycholate, 150 mM NaCl, 1 mM EDTA pH 8.0, 1 mM PMSF, Pierce™ Protease& Phosphatase Inhibitor Mini Tablets) and final protein concentration was determined with BCA protein Assay (Biorad Laboratories, Feldkirchen, Germany). Heart tissue samples of the infarct areas were excised (2- or 8 weeks post-CI) using a macroscope equipped with fluorescence (Axio Zoom V16, Zeiss). For protein isolation, heart tissue was homogenized (with micro pellet pestle (Wilmad LabGlass (Vineland, NJ, USA)), at 4 °C) using RIPA buffer (see above) and protein concentration was determined as described above. For gel electrophoresis, SDS mini gels (8% or 12% separating gel: 8% or 12% Acrylamide, 7.5 mM Tris-HCL (pH 8.8), 0.1% SDS, 0.05% APS, 0.01% TEMED; 4% or 8% stacking gel: 4% or 8% Acrylamide, 1.25 mM Tris-HCL (pH 6.8), 0.1% SDS, 0.05% APS, 0.01% TEMED) were prepared, proteins diluted in 2x or 4x Laemmli buffer (Biorad) (for 2x Laemmli: 1:2 dilution, for 4x Laemmli: 1:4 dilution) to 20 µg and denaturated for 10 min at 100°C. Proteins were separated by electrophoresis (ProSieve EX Running buffer 10x, diluted in H_2_O to 1x, (Lonza, Basel, CH); Precision Plus Protein WesternC Standards, Biorad). Then, separated proteins were blotted onto PVDF-membranes (Methanol activated, Low fluorescence, 0.2 µm (Biozym Scientific GmbH, Hessisch Oldendorf, Germany) by tankblot for 75 min and 100 V (ProSieve EX Transfer buffer 10x, diluted in H_2_O to 1x, Lonza). Membranes were blocked with 5% milk powder (MP, Skim milk powder, (VWR, Radnor, PA, US)) in 1x TBST buffer (20 mM Tris, 150 mM NaCl, 0.1% Tween-20 in H_2_0) for minimum 1 h. Antibodies, diluted in 5% MP/TBST, (Cx43: 1:3000 custom-produced rabbit polyclonal; eGFP: 1:1000 mouse monoclonal (Clontech Laboratories Inc, Mountain View, CA, US); αSMA: 1:1000 mouse monoclonal, Merck; Vimentin: 1:1000 rabbit monoclonal (Cell signalling); Periostin: 1:1000 rabbit polyclonal (Novus Biologicals, Centennial, CO, US); TGFb1: 1:500 rabbit polyclonal (Abcam, Cambridge, UK) were incubated overnight at 4°C. As housekeeper, Horseradish peroxidase (HRP)-conjugated GAPDH (1:5000, Sigma Aldrich) or ß-Actin-HRP (1:10000, Sigma-Aldrich) was incubated for 1 h at RT and in 5% MP/TBST. Followed by 3 washing steps with 1x TBST, secondary antibodies (donkey-anti-rabbit Alexa Fluor 488: 1:3000; donkey-anti-mouse IGg2a Cy5: 1:3000; Affinipure, Jackson Immuno Research) and Precision Protein StrepTactin-HRP Conjugate (1:3000, Biorad), diluted in 5% MP/TBST, were incubated for 1 h at RT. Signals were developed with Pierce ECL Western blotting Substrate (Thermo Fisher) and detected with ChemiDoc MP Imaging System (Biorad). Quantification of target protein expression was performed with Image Lab Software (Biorad) and normalised to GAPDH or ß-Actin expression.

### Approval of animal experiments and study design

All mouse experiments were performed in accordance with the ARRIVE guidelines, the guidelines of the German law of protection of animal life, and approved by the local government authorities (Landesamt für Natur- und Verbraucherschutz Nordrhein-Westfalen, NRW, Germany); animal protocol numbers: 81-02.04.2018.A120 and 81-02.04.2020.A093. Even before the authorities approved the animal protocol, it was first determined whether the “3 R rules - Replace, Reduce, Refine” were implemented in our experimental design. We have used several control groups to prove the effectiveness of our therapy: The cryoinfarction (CI (only)) group to assess the extent and cellularity of the cardiac scar and its functional effects over time, the LV-eGFP control group (CI + injection of LV-transduced mFB) to investigate the possible effects of the transplantation of LV-transduced mFB on cardiac pump function and electrophysiological properties *in vivo*, and to compare these with the LV-Cx43-eGFP group. We have also performed sham operations (thoracotomy and pericardial incision) to assess possible side effects of the surgical procedure.

### Statistics

Statistical evaluation of data with more than 2 groups was performed using one-way ANOVA with post-hoc Tukey test. Data sets consisting of only 2 groups were analysed employing Student's t-test. Electrophysiological data were compared using a Fisher's exact test. Normal distribution was tested by Shapiro-Wilk test (n < 50). A p value < 0.05 was considered statistically significant, error bars represent SEMs.

## Results

### Characterisation of PMAO-MNP in fibroblasts

**Figure 1:**
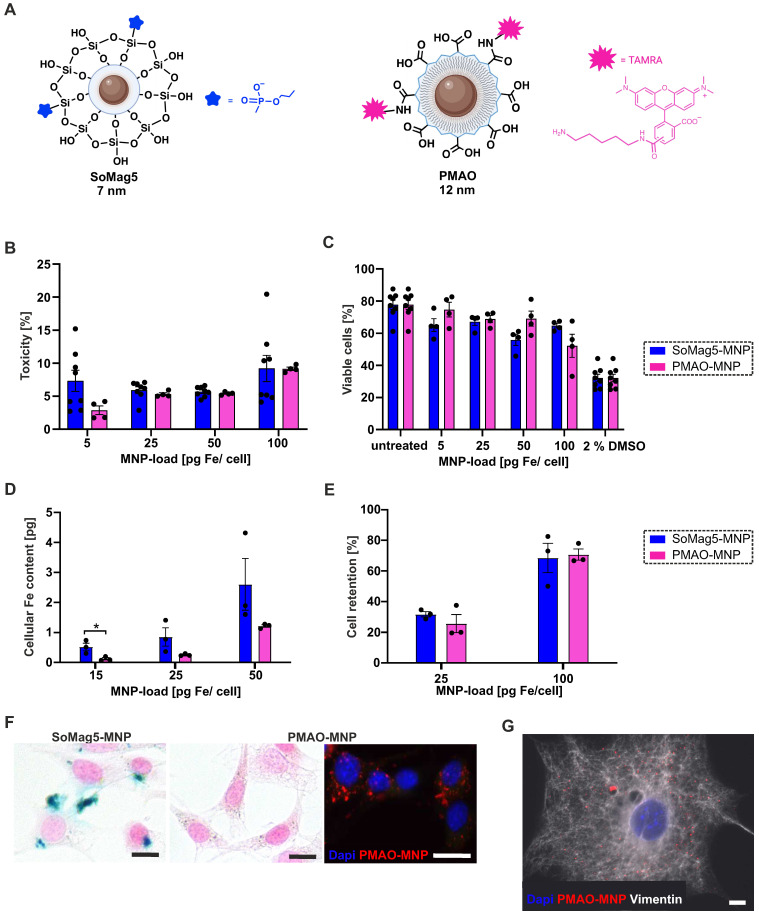
Toxicity, magnetic potential, and intracellular distribution of SoMag5- and PMAO-MNP in 3T3-fibroblasts (3T3-FB). (A) Schemes of the two tested MNP. (B) LDH-toxicity- and (C) MTT-viability-assays of MNP 24 h after MNP loading without magnet application for 1 h. (D) Magnetic particle spectrometry (MPS) of MNP-loaded cells. (E) Cell retention of MNP-loaded cells assessed by using a magnet rack. (F) Prussian blue iron staining of PMAO- and SoMag5-loaded 3T3-FB (25 pg Fe/cell: blue: iron oxide, red: nuclei, bar: 5 µm; fluorescence macroscopic image: blue: nuclei, red: PMAO-MNP, bar: 20 µm). (G) Microscopic image of a 3T3-FB incubated overnight with PMAO-MNP (25 pg Fe /cell, red: PMAO-MNP, blue: nuclei, white: Vimentin, bar: 10 µm). Data are presented as the mean ± SEM. P-value: * < 0.05**.** Figure 1A was created with BioRender.com. Abbreviations: fibroblasts (FB); magnetic nanoparticles (MNP).

In our earlier study, we showed strongly improved engraftment rates of eCM when the core-shell silica-iron oxide magnetic nanoparticles SoMag5-MNP were combined with magnetic steering 10. Since SoMag5-MNP tend to form cytosolic aggregates, we have tested a new type of MNP with a larger iron core (12 *vs* 7 nm diameter) but a smaller hydrodynamic diameter compared to SoMag5-MNP (23 *vs* 40 nm) (Figures 1A, S1A,B). These MNP are obtained by thermal decomposition of iron acetylacetonate, a method that provides monodisperse nanoparticles with uniform size and well-defined shape, in contrast to the co-precipitation method used to obtain SoMag5-MNP. The MNP were coated with an amphiphilic polymer (poly maleic anhydride-alt-1-octadecene), PMAO) to yield PMAO-MNP. An additional advantage is their fluorescent surface by labelling with a fluorophore (5-carboxytetramethylrhodamine, TAMRA), as the MNP can be detected in the tissue after intra-organ injections. First, we investigated and compared the toxicity and the magnetic potential between PMAO- and SoMag5-MNP in NIH-3T3 fibroblasts (3T3-FB) *in vitro*. LDH toxicity analysis showed similar values for both types of particles (Figure 1B). The MTT assay (Figure 1C) yielded similar results, as 69% and 67% viable cells were found at 25 pg Fe/cell for PMAO- and SoMag5-MNP, respectively. An increase in iron concentration (100 pg Fe/cell) resulted in increased toxicity in 3T3-FB, as the viability of cells decreased to 65% and 52% for SoMag5- and PMAO-loaded cells, respectively (Figure 1C). To determine the cellular Fe uptake in FB, we measured the nanoparticular Fe content of 3T3-FB using magnetic particle spectrometry (MPS), revealing that cellular uptake of PMAO-MNP was only slightly lower compared to SoMag5-MNP (Figure 1D). We also assessed the magnetic potential of the MNP-loaded cells in a metal rack. We found similar retention rates in a magnetic field irrespective of slight differences in cellular MNP uptake (Figure 1E). Importantly, Prussian blue iron staining confirmed earlier reports 10 that SoMag5-MNP (Figure 1F, left panel), but not PMAO-MNP (Figure 1F, right panel), formed intracellular aggregates that could have adverse cell biological effects. When investigating the intracellular distribution pattern of PMAO-MNP using their fluorescence due to the TAMRA-fluorochrome linked to the polymer coating (Figure 1F, right picture, Figure S1C), a homogenous intracellular distribution pattern was observed in 3T3-FB (Figure 1G). As PMAO-MNP displayed good uptake into 3T3-FB, high cell retention rates, low toxicity, and no cytosolic aggregate formation, we used these particles for all the following experiments.

### Characterisation of transduced mFB

**Figure 2:**
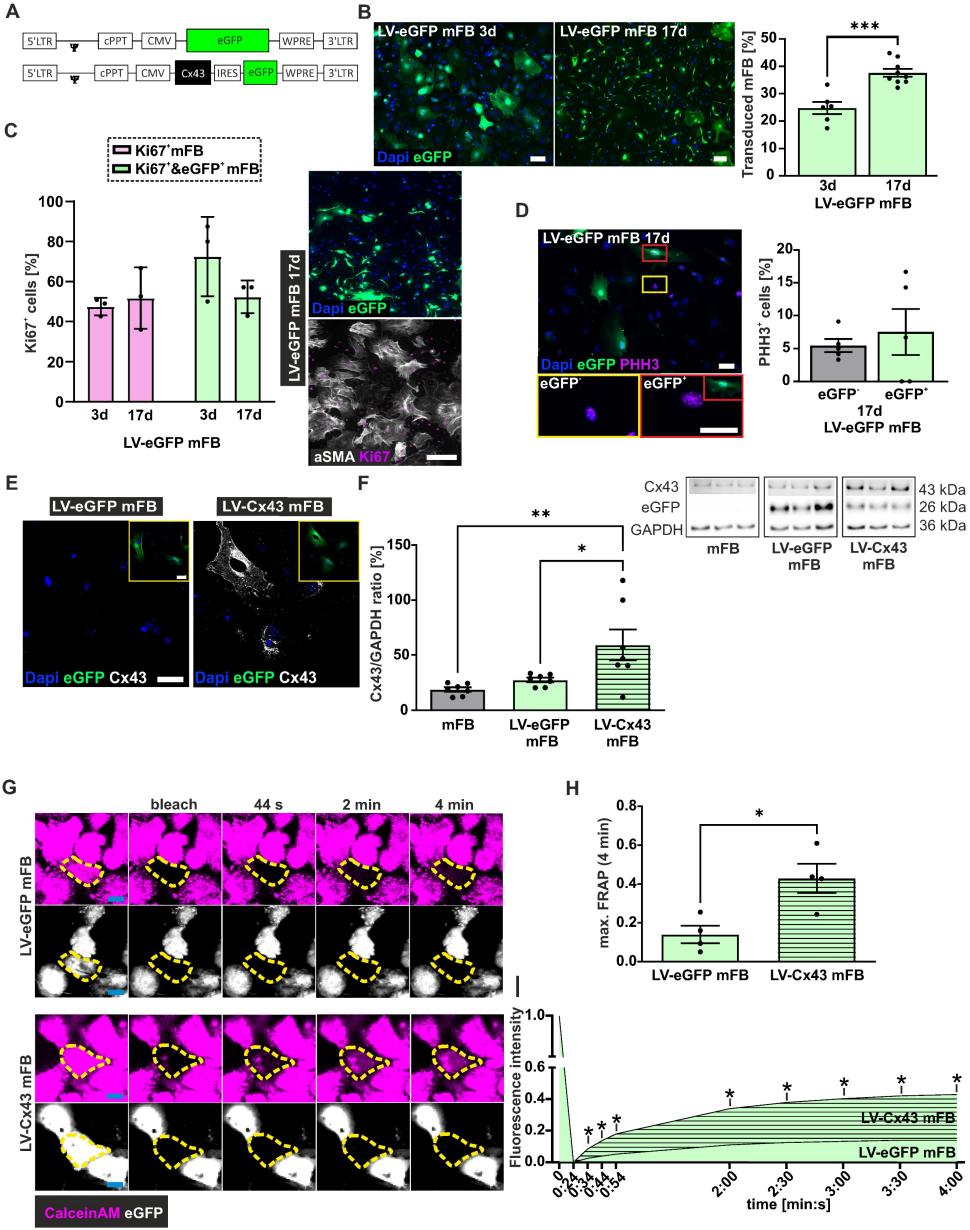
LV-based transduction of embryonic cardiac myofibroblasts (mFB) *in vitro*. (A) LV-constructs (MOI = 5) used for the transduction of mFB. (B) Representative fluorescence pictures and percentage of eGFP^+^ mFB 3 and 17 days after LV transduction (bars: 50 µm). (C) αSMA and Ki67 co-stainings of LV-transduced mFB 17 days post-transduction; quantitation of Ki67^+^/eGFP^+^ mFB 3- and 17 days post LV-eGFP transduction (bar: 200 µm). (D) PHH3 staining of mFB 17 days post LV-eGFP transduction (bar: 50 µm). (E) Cx43 overexpression in mFB 3 days after LV transduction (white: Cx43, blue: nuclei, green: eGFP, bar: 50 µm). (F) Cx43 protein expression using Western Blotting in mFB 3 days after LV transduction of mFB. (G) Representative images of FRAP in mFB 6 days after transduction with LV-eGFP or LV-Cx43 (violet = Calcein AM Violet, white = eGFP, bar = 20 µm). (H) Quantitation of max FRAP after 4 min and (I) fluorescent recovery over time (n = 4). Data are presented as the mean ± SEM. P-values: * < 0.05; ** < 0.01; *** < 0.001**.** Abbreviations: Connexin 43 (Cx43); days (d); fluorescent recovery after photobleaching (FRAP); lentivirus (LV); myofibroblasts (mFB).

For grafting experiments, mFB were harvested from E13.5 mouse hearts, propagated, and enriched in cell culture. As reported before 24-26, molecular and cell biological assays revealed significantly increasing alpha smooth muscle actin (αSMA) levels during cell culture in accordance with their differentiation from cardiac FB into αSMA^+^ mFB (Figure S1D,E). At the time of transplantation > 90% of cells stained positive for αSMA and the FB marker PDGFRα (Figure S1F); LV transduction of cells had no effect on the αSMA expression (Figure S1G). For transduction, we used two LV, as reported before 7, one harbouring a bicistronic expression cassette for Cx43 and eGFP (LV-Cx43) and the control virus with eGFP only (LV-eGFP) (Figure 2A). mFB were transduced overnight at 8 days after cell isolation with LV (Figure 2A, MOI = 5, Figure S1D).

Transduction efficiency could not be determined using flow cytometry, as the cells did not tolerate the enzymatic digestion, and we therefore assessed it using fluorescence microscopy. At 3 days after transduction, approximately 25% of cells were eGFP^+^ using either LV (Figure 2B); the eGFP fluorescence was, as reported earlier, weaker when using the Cx43 LV because of the bicistronic vector construct 20. We determined the transduction rate (eGFP^+^ cells) also at 17 days after LV treatment and found with ~ 38% a clearly higher ratio of eGFP^+^ cells (p-value = 0.0002); this time point is more relevant for estimating the engraftment of cells upon intracardiac injections (Figure 2B). This increase could be due to augmenting expression levels of eGFP over time or a greater proliferation rate of eGFP^+^ mFB. Co-staining against Ki67 and αSMA at 17 days post-transduction revealed that ~ 50% of all mFB were Ki67^+^ (48% at day 3, 52% at day 17). When analysing eGFP^+^ mFB only, the ratio of Ki67^+^/eGFP^+^ was even higher at day 3 (~ 72%), whereas there was no difference at day 17 (~ 52%) (Figure 2C). Staining against the M-phase marker Phospho-histone H3 (PHH3) confirmed the strong proliferation rate of mFB (6.6% native *vs* 7.5% LV-transduced mFB PHH3^+^, Figure 2D).

Next, we analysed Cx43 protein expression upon LV-Cx43 transduction of mFB using immunostaining and found that the Cx43 signal was distributed throughout the cell membrane and that it was clearly increased 3 days after transduction (Figure 2E). This was confirmed by Western blotting, in which an approximately 2-fold increase of Cx43 protein expression in LV-Cx43 transduced mFB compared to LV-eGFP and not-transduced mFB was found (Figure 2F, p-values = 0.035 and 0.0073).

We also investigated the formation of functional gap junctions in mFB following LV-Cx43 transduction with fluorescence recovery after photobleaching (FRAP). The cells were loaded with the gap junction-permeable dye Calcein AM violet, and individual cells within cell clusters bleached with laser light. The Calcein AM violet influx from neighbouring cells (Figure 2G,H,I) was measured over 4 min and found to be significantly faster in LV-Cx43 compared to LV-eGFP transduced cells throughout the whole observation period (max 3-fold after 4 min (p-value = 0.016), confirming the formation of functional Cx43 gap junctions.

Furthermore, we assessed the consequences of Cx43 overexpression in mFB by analysing the transcriptome of LV-Cx43 and LV-eGFP (n = 3) transduced mFB after 3 days (Figure S2). Bioinformatic analysis revealed that 697 genes were up- and 702 downregulated in LV-Cx43 compared to LV-eGFP mFB (Figure S2A). Gene clustering and gene ontology analysis showed that the most strongly downregulated genes in LV-Cx43 mFB are related to immune response pathways and apoptotic processes, whereas strongly upregulated genes in LV-Cx43 mFB are clustered in angiogenesis and heart development gene groups (Figure S2C,D,I). When looking at specific developmental and cardiogenic genes, such as *tnnt2*, *gata4*, and *atp2a2* we observed an enrichment in LV-Cx43 mFB, likewise also ion channels usually found in CM and angiogenesis/endothelial cell marker genes, such as *flt1*, *pecam1*, *gata4* and *hspg2* (Figure S2B). The data were confirmed by qPCR analysis, proving that *tnnt2*, *gata4* and *atp2a2* were indeed upregulated (Figure S2F-H). Importantly, the transduced cells retained their mFB phenotype, as they stained positively for mFB markers such as αSMA but not CM markers such as cTroponin (Figure S1E,F).

Thus, mFB show a transduction rate of approximately 40% using LV, the cells overexpress Cx43 and display high proliferation rates.

### Grafting of LV-transduced mFB into the cardiac lesion

**Figure 3:**
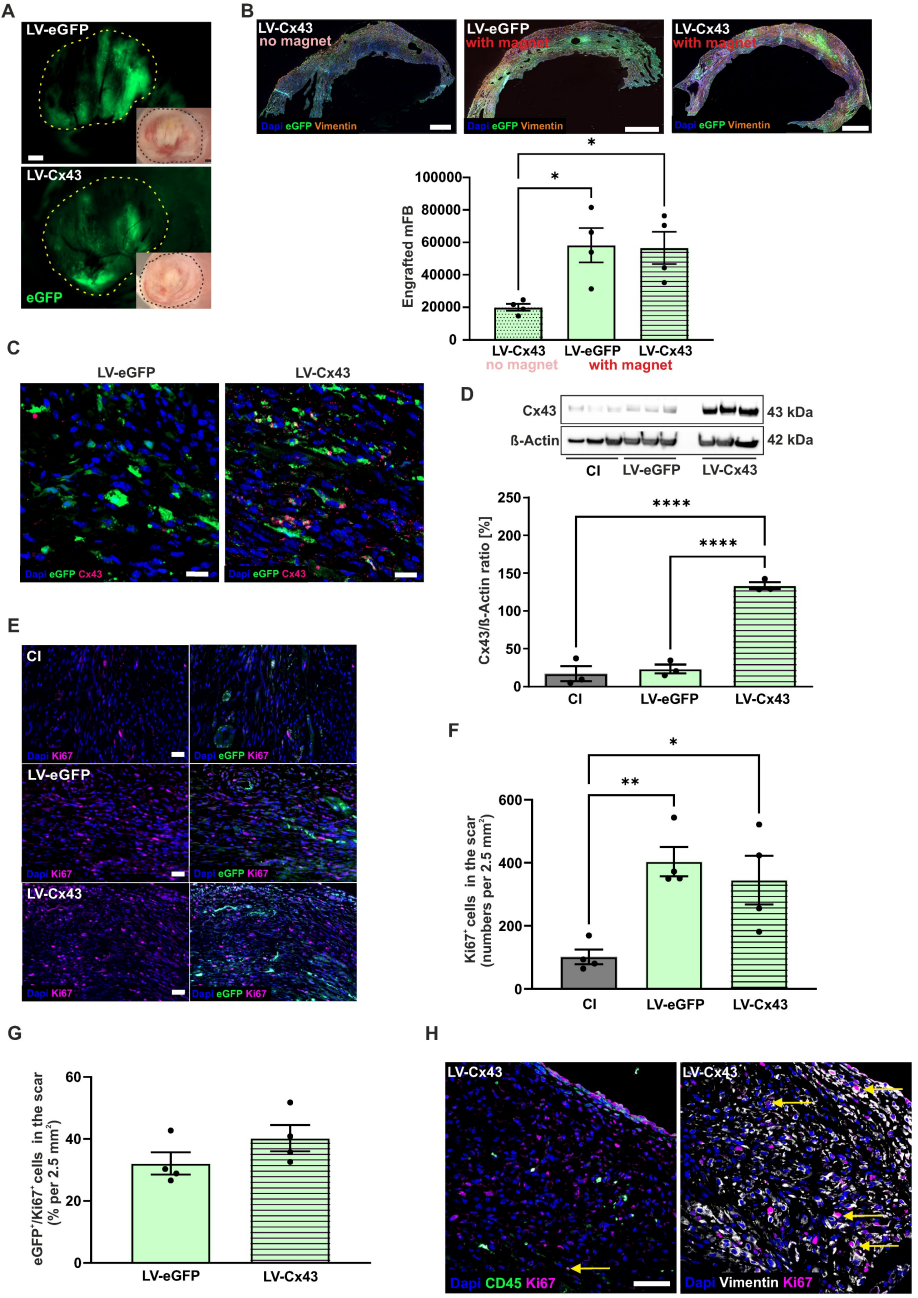
mFB engraftment and Cx43 expression in the cardiac scar of mice 2 weeks post-CI and intracardiac injection of LV-transduced mFB. (A) Macroscopic images of mFB injected hearts (green: native eGFP fluorescence) (dots mark the scar area, bars: 500 µm). (B) Quantitation of engrafted eGFP^+^ mFB in heart sections stained against eGFP and Vimentin, representative pictures are shown (right panels, bar: 500 µm). (C) Immunostainings against Cx43 in LV-eGFP and LV-Cx43 heart sections (bars: 20 µm). (D) Quantitation of Cx43 protein expression in cut out scars with Western blotting. (E) Immunostainings against Ki67 and eGFP in scar areas of CI, LV-eGFP and LV-Cx43 heart sections (bar: 50 µm) (F) Quantitation of total Ki67^+^ cells and (G) percentage of eGFP^+^/Ki67^+^cells in CI, LV-eGFP and LV-Cx43 heart sections. (H) Co-immunostainings against CD45/Ki67 or Vimentin/Ki67 (bar: 50 µm) in cardiac scars of LV-Cx43 heart sections (arrows indicate CD45/Ki67^+^ or Vimentin/Ki67^+^ cells). Data are presented as the mean ± SEM. P-values: * < 0.05; ** < 0.01; **** < 0.0001. Abbreviations: cryoinjury (CI); Connexin 43 (Cx43); lentivirus (LV); myofibroblasts (mFB).

Next, engraftment properties and cell biological consequences of injecting LV-transduced and MNP-loaded cardiac mFB into acute CI *in vivo* were assessed. 2.0 x 10^5^ LV-transduced and PMAO-MNP-loaded mFB (Figure S1C) were injected into cryoinfarcted (CI) wild-type CD1 mice (5 µl per lesion). This injury type was used as it is highly reproducible and best suited for assessing the functional impact of cell therapy 7, 31. To increase cell engraftment, we used magnetic steering by positioning a rod magnet (1.3 T) at 5 mm distance from the surface of the heart during and for 10 min after injection of the MNP-loaded mFB 10. Three control groups with CI were used: mice with CI, with CI/injection of LV-eGFP mFB (LV-eGFP)/with magnet), and with CI/injection of LV-Cx43 mFB (LV-Cx43/no magnet). Furthermore, sham-operated mice (thoracotomy and incision of the pericardial sack) were generated as healthy controls for functional experiments. At 2 weeks post-CI, hearts were harvested and processed for analysis. As shown in Figure 3A, using a macroscope, engrafted mFB could be identified based on native eGFP fluorescence. Engraftment rates were estimated by counting eGFP^+^ mFB in heart sections using a fluorescence microscope (Figure 3B). When using magnetic steering, approximately 35.000 - 82.000 eGFP^+^ mFB were counted in the scar area (average = 57.620 ± 6.638 cells, n = 8). These numbers are 4-fold higher compared to injecting 2.0x10^5^ eCM 10, even though in the present case only 40% of injected cells were eGFP^+^. We next determined the impact of magnetic steering by counting the Vimentin^+^/eGFP^+^ cells in infarcted LV-Cx43 hearts either with or without magnet application at 2 weeks post-CI. We found that it increased grafting ~ 3-fold (Figure 3B, p-value = 0.025 and Figure S3A-C). We also analysed Cx43 expression in the cardiac scar, immunohistochemistry revealed strong cellular Cx43 expression in sections from LV-Cx43 hearts compared to those from LV-eGFP injected animals (Figure 3C). This was underscored by Western blotting analyses of excised scar tissue, in which a ~ 5 - 6-fold increase of Cx43 protein content compared to control hearts was detected (CI, LV-eGFP; both p-values < 0.0001; Figure 3D), proving efficient transduction *in vitro* and implying prominent cell engraftment.

To explore, whether proliferation of injected mFB could contribute to prominent engraftment rates we co-stained sections of LV-eGFP, LV-Cx43 and control hearts against Ki67 and eGFP 2 weeks post-CI (Figure 3E). We detected significantly increased numbers of Ki67^+^ cells in the cardiac scar upon mFB transplantation (cells/2.5 mm² area: LV-eGFP: 403, p-value = 0.0084; LV-Cx43: 345, p-value = 0.027; for both n = 4) compared to CI (102, n = 4, Figure 3F). When counting eGFP^+^ mFB only, we found ~ 36% Ki67^+^ cells (Figure 3G). As only ~40% of injected mFB were eGFP^+^, the interpretation of the data was difficult, as eGFP^-/^Ki67^+^ cells could be either transplanted or endogenous FB. To rule out that increased numbers of Ki67^+^ cells upon mFB injection are due to immune cell infiltration we triple-stained against CD45, Vimentin, and Ki67 and found that only a very low number of CD45^+^/ Ki67^+^ cells (Figure 3H).

Taken together these data demonstrate prominent engraftment and proliferation of mFB in the cardiac scar when combining MNP loading with magnetic steering.

### Grafting of genetically labelled neonatal mFB (exoFB) into the cardiac lesion

**Figure 4:**
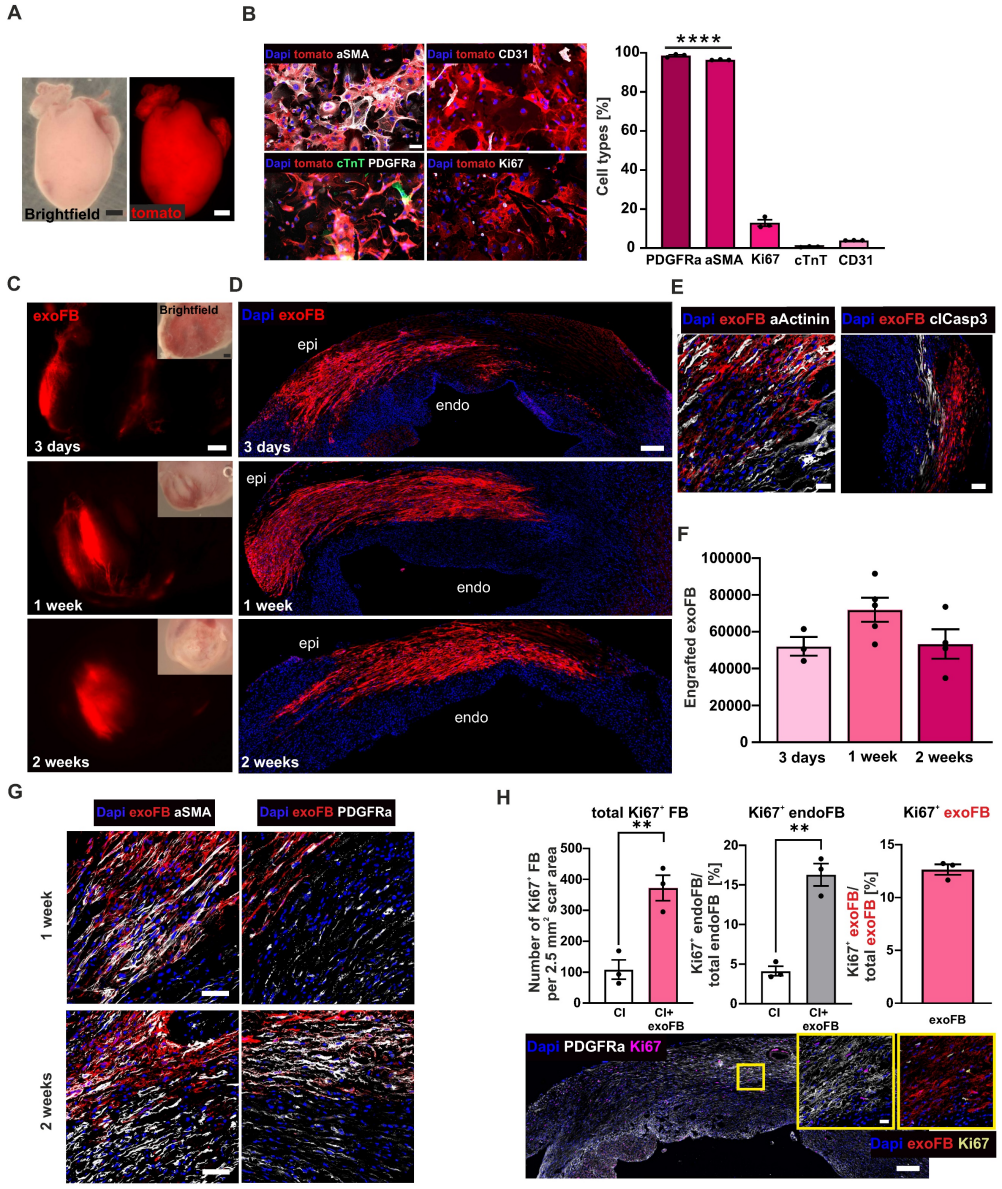
Cell biological characterisation of exoFB, engraftment properties of exoFB at different time points post-CI. (A) Macroscopic images of tomato^+^ P3 heart (red: tomato; bar: 500 µm). (B) Cell biological characterisation and quantitation of isolated cardiac cells after 12 days in cell culture using immunostainings against different cellular markers (blue: nuclei, red: exoFB, green: cardiac TroponinT, white: PDGFRα/ αSMA/ Ki67/ CD31, bar: 50 µm). (C) Macroscopic images of cardiac scars with grafted exoFB 3 days, 1- and 2 weeks post-CI (bar: 500 µm). (D) Overview of the distribution pattern of exoFB 3 days, 1- and 2 weeks post-CI in the scar (blue: nuclei, red: exoFB, bar: 200 µm). (E) Images showing apoptotic CM (αActinin^+^/clCasp3^+^) in-between exoFB 1week post-CI (blue: nuclei, red: exoFB, white: αActinin/clCasp3, bar: 20 and 100 µm). (F) Quantitation of engrafted exoFB; for the analysis tomato^+^ exoFB (n = 9) and tomato^+^/eGFP^+^ exoFB (after tamoxifen treatment, n = 3) were counted. (G) Characterisation of FB maturation in the scar of hearts 1- and 2 weeks post-CI and exoFB injection using immunostainings. Markers: αSMA, PDGFRα (blue: nuclei, red: exoFB, white: αSMA/ PDGFRα, bar: 20 µm). (H) Amount of proliferating FB in CI control hearts vs exoFB-injected hearts 2 weeks post-CI (left graph). Percentage of Ki67^+^ endoFB of total endoFB/area (middle graph) and percentage of Ki67^+^ exoFB of total exoFB/area (right graph) 2 weeks post-CI. Representative picture of an exoFB injected heart 2 weeks post-CI, higher magnification shown in insets (left: blue: nuclei, white: PDGFRα, magenta: Ki67, bar: 200 µm; right: blue: nuclei, red: exoFB, yellow: Ki67, bar: 20 µm). Data are presented as the mean ± SEM. P-values: ** < 0.01; **** < 0.0001. Abbreviations: cardiomyocytes (CM); cryoinjury (CI); cardiac TroponinT (cTnT); endogenous fibroblasts (endoFB); neonatal cardiac myofibroblasts (exoFB); fibroblasts (FB).

Given the apparently strongly increased engraftment numbers compared to other cell types, and the fact that only 40% of the injected cells were eGFP labelled, we employed another approach to corroborate these findings. We used genetically labelled neonatal mFB (exoFB, Figure 4A, for details see Methods section) and found that after 12 days in cell culture > 95% of cells were exoFB (αSMA^+^, p-value < 0.0001), whereas percentages of endothelial cells (CD31^+^~ 4%) and CM (cTnT^+^~ 0.7%) were negligible and comparable to earlier time points of cultivation (Figures 4B, S4A). The percentage of Ki67^+^ exoFB in cell culture was 13% (Figure 4B), indicating that the proliferation rate of postnatal exoFB was lower than that of embryonic mFB.

Intracardiac injections of exoFB were performed, as described. All hearts were analysed (without macroscopic preselection) at 3 days, 1- and 2 weeks (Figure 4C) post-CI. Quantitation of engrafted exoFB yielded 52.059 ± 5.062 cells (n = 3) at 3 days, 71.934 ± 6.536 cells (n = 5) at 1 week and 53.312 ± 7.938 cells (n = 4) at 2 weeks (Figure 4F) post-CI corroborating the very good engraftment rates obtained using mFB. We next investigated the distribution pattern of the grafted exoFB (tomato^+^) in the scar, their migration and interaction with endogenous FB (endoFB, tomato^-^). 3 days and 1-week post-CI exoFB were sticking together, located in between dying CM and close to the epicardial layer (Figure 4D). Only at this early time point (1 week) a few CD45^+^ or tomato^+^/ CD31^+^ cells interspersed between the FB populations were found (Figure S4B). Akin to our findings with mFB, we also investigated apoptosis and detected cardiac α-actinin^+^/caspase3^+^ CM but very few caspase3^+^ endo- and exoFB (Figure 4E). 2 weeks post-CI, exoFB remained close to the epicardium, but were sticking closer together (Figure 4D) and remained clearly separate from endoFB. The exoFB remained αSMA^+^ and Vimentin^+^ upon grafting, but underwent further differentiation and maturation, as immunostainings showed decreasing αSMA and increasing PDGFRα levels (Figure 4G), as observed by other groups for resident FB in the cardiac scar 12, 32; 2 weeks post-CI neither tomato^+^ inflammatory nor endothelial cells were detected (Figure S4B). Interestingly, we noticed that the number of engrafted exoFB and mFB at 2 weeks post-CI was very similar, even though only 40% of mFB were eGFP-labelled. We therefore assessed their proliferation rate in heart sections at 2 weeks post-CI using Ki67 staining. In accordance with the *in vitro* data was the proliferation rate of exoFB with 13% (Figure 4H, right graph) significantly lower (p-value = 0.0012) than that of mFB (36%), explaining the observed differences. Interestingly, tomato^+^ exoFB and tomato^-^/PDGFRα^+^ endoFB displayed significantly higher proliferation rates upon cell grafting than CI controls (Figure 4H, left graph; p-value = 0.007). In fact, transplanted exoFB increased endoFB proliferation 4-fold, as 16% of tomato^-^ endoFB were Ki67^+^ *vs* only 4% in CI controls 2 weeks post-CI (Figure 4H, middle graph; p-value = 0.0013).

Thus, exoFB stably integrate into the cardiac scar, and further proliferate and differentiate.

### Functional impact of grafting Cx43-overexpressing mFB into the cardiac lesion 2- and 8 weeks post-surgery

**Figure 5:**
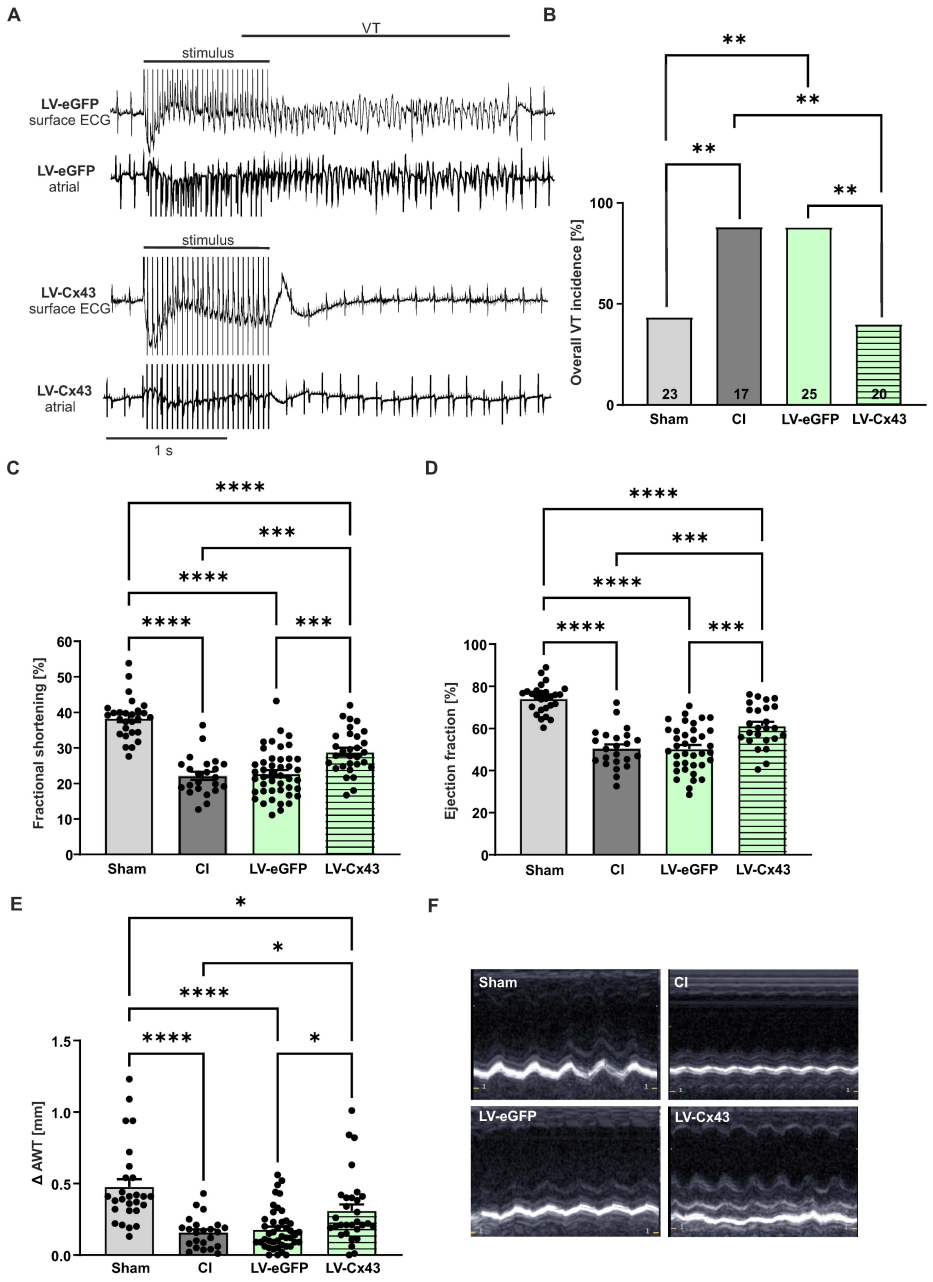
Electrophysiological testing *in vivo* and echocardiographic assessment of left ventricular function 2 weeks post-CI. (A) Representative surface ECG traces of a LV-eGFP (upper traces) and a LV-Cx43 (lower traces) mouse; note the capture and self-terminating VT in the LV-eGFP mouse, the post-stimulation pause and ensuing sinus rhythm in the LV-Cx43 mouse upon burst stimulation. (B) Quantitation of VT incidence in the different groups (n numbers: sham = 23; CI = 17; LV-eGFP = 25; LV-Cx43 = 20). (C) Fractional shortening, (D) ejection fraction and (E) Δ anterior wall thickening (Δ AWT in mm). (F) Pictures represent original M-mode recordings of the different groups. Data are presented as the mean ± SEM. P-values: * < 0.05; ** < 0.01; *** < 0.001; **** < 0.0001. Abbreviations: Δ anterior wall thickening (Δ AWT), cryoinjury (CI); Connexin 43 (Cx43); electrocardiogram (ECG); lentivirus (LV); myofibroblasts (mFB); ventricular tachycardia (VT).

Given the prominent engraftment characteristics of mFB into the cardiac scar we tested whether also its functional properties could be modulated with this approach. We overexpressed Cx43 in mFB *in vitro* using the LV-Cx43 vector and grafted 2 x 10^5^ cells into the cardiac lesion, as earlier results of our group demonstrated that Cx43 overexpression can reduce post-infarct VT incidence *in vivo* 7, 11. To assess arrhythmia vulnerability, *in vivo* electrophysiological testing was performed using S1-S2 and burst stimulation protocols 2 weeks post-CI. In Figure 5A, representative traces of surface and intracardiac atrial ECG leads for control (LV-eGFP) and Cx43 (LV-Cx43) mice showed that burst stimulation (Fig 5A, upper leads) induced a self-terminating VT in control hearts, whereas no VT were typically evoked in the LV-Cx43 hearts (Fig 5A, lower leads). The original traces evidenced ventricular capture and atrioventricular (AV) dissociation during burst pacing in a representative LV-Cx43 heart and a normo-frequent sinus rhythm after a short compensatory pause after electrical pacing (Fig 5A, lower leads). CI and LV-eGFP control mice displayed a high VT incidence (88%), which was strongly reduced to 40% in LV-Cx43 mice (CI *vs* LV-Cx43, p-value = 0.0055; LV-eGFP *vs* LV-Cx43, p-value = 0.0012) in the range of non-infarcted controls (Sham: 43%, Figure 5B).

Cardiac pump function was determined by echocardiography 13 days post-CI (Figure 5C-F). All infarcted animals (CI, LV-eGFP, LV-Cx43) showed, as expected, a significant reduction in fractional shortening, (FS, Figure 5C), ejection fraction (EF, Figure 5D) and anterior wall thickening (Δ AWT, Figure 5E) compared to shams. Surprisingly, LV-Cx43 mice displayed a significant improvement in left ventricular pump function (FS = 28.9%, EF = 61.3%) compared with CI (FS = 22.2%, p-value = 0.0007; EF = 50.5%, p-value = 0.0009) and LV-eGFP mice (FS = 22.7%, p-value = 0.0003; EF = 50.4%, p-value = 0.0001), while heart rates were similar in all three groups (data not shown). Also, Δ AWT (0.31 mm) was significantly improved in LV-Cx43 mice compared to CI (0.16 mm, p = 0.035) and LV-eGFP (0.18 mm, p = 0.032) mice, respectively. To exclude that the variation in LV function was due to different infarct sizes, quantitative morphometry was performed, which did not evidence prominent differences in infarct area or volume between the groups (Figure S6A,B).

**Figure 6:**
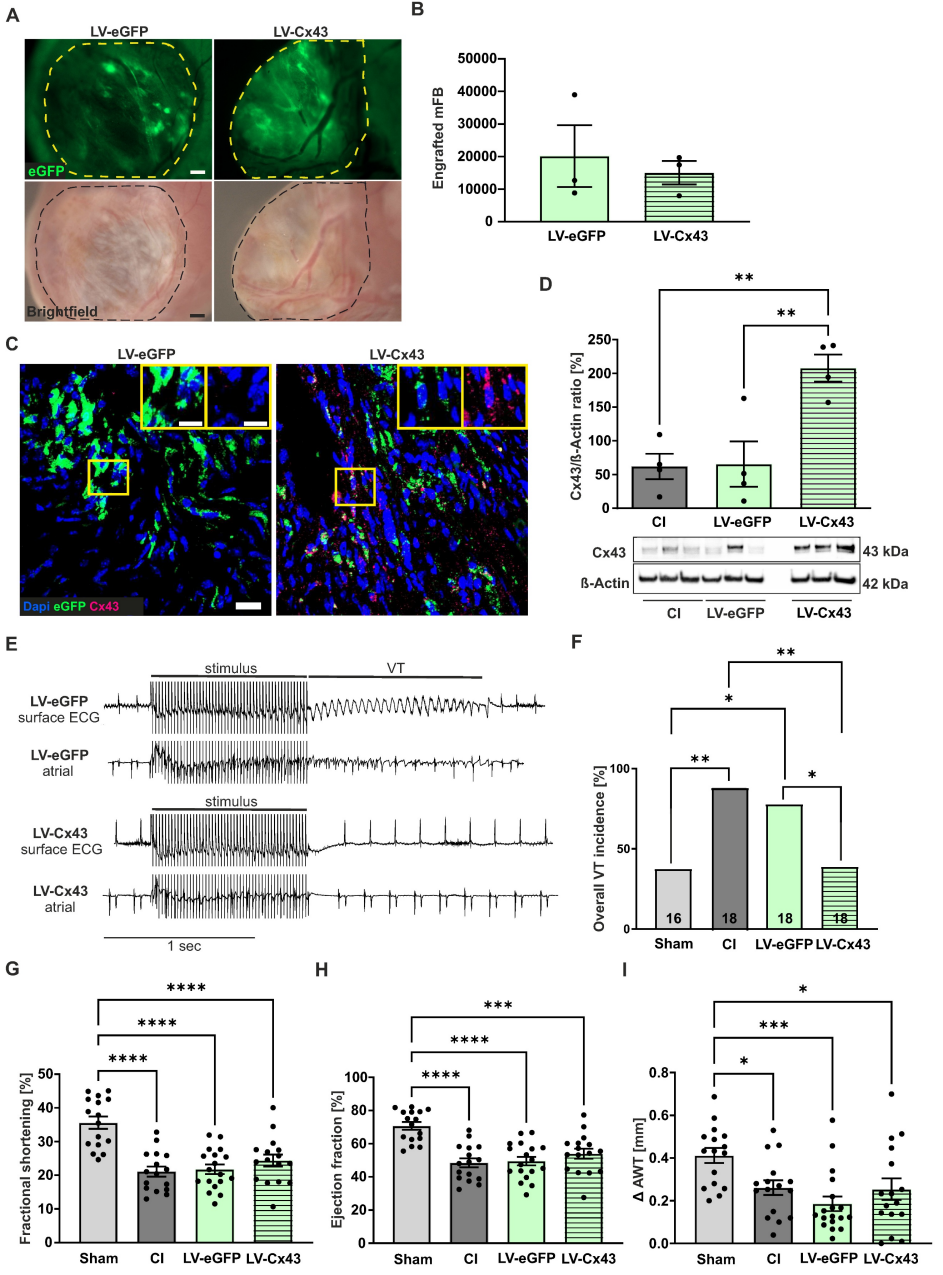
Long-term engraftment of mFB and functional impact 8 weeks post-CI. (A) Macroscopic pictures documenting engraftment of eGFP^+^ mFB in the scar area (dotted area, bars: 500 µm). (B) Quantitation of engrafted eGFP^+^/Vimentin^+^ mFB. (C) Anti-eGFP and Cx43 stainings of mFB in sections of a LV-eGFP and a LV-Cx43 heart (blue: nuclei, green: eGFP, pink: Cx43, bars: 20 µm and 5 µm). (D) Cx43 protein expression in cut-out scars analysed by Western blotting. (E) Representative surface ECG traces and (F) VT incidence in the different groups of mice (n numbers: sham = 16; CI = 18; LV-eGFP = 18; LV-Cx43 = 18). Echocardiographic assessment of fractional shortening (G), ejection fraction (H) and Δ anterior wall thickening (I) in the different groups of mice. Data are presented as the mean ± SEM. P-values: * < 0.05; ** < 0.01; *** < 0.001; **** < 0.0001. Abbreviations: Δ anterior wall thickening (Δ AWT), cryoinjury (CI); Connexin 43 (Cx43); electrocardiogram (ECG); lentivirus (LV); myofibroblasts (mFB); ventricular tachycardia (VT).

We also investigated the functional impact 8 weeks post-CI, as it is known that grafted cell numbers in the infarcted mouse heart strongly decrease over time 9, 10. At this stage, we still detected with a macroscope eGFP^+^ patches in the scar, indicating successful long-term engraftment of mFB (Figure 6A). We selected LV-eGFP and LV-Cx43 hearts with proven engraftment under the macroscope and found 8.000 to 39.000 (average= 17.605 ± 4.670, n = 6) eGFP^+^ mFB (Figure 6B). Despite strongly improved engraftment rates using mFB and exoFB, our data revealed a steady cell loss over time, and we, therefore, investigated the potential mechanisms. The cardiac scar area is known to become thinner and less cellular over time, and we quantified this and found both, a reduction of the wall thickness (334 ± 23.59 µm at 2 weeks *vs* 222 ± 11.98 µm at 8 weeks; p-value = 0.016; both n = 3) and of cellularity in the border zone and the centre of the lesion (Figure S5A). The decrease in wall thickness in the long-term course also explains the morphometric decrease of the infarct volume (Figure S6B,D) despite comparable infarct surfaces (Figure S6A,C). Because neither strong immune cell infiltration nor apoptosis of grafted cells was detected (Figure S5C), we characterised the cell biological characteristics of the mFB using immunostainings. We found that the transplanted mFB and exoFB displayed typical features of activated myofibroblasts given their strong αSMA expression 1- and 2 weeks post-CI and mFB injection, whereas after 8 weeks, the cells had lost αSMA and displayed strong Tcf21 expression (Figure S5B), consistent with their transdifferentiation into a senescent state, namely into matrifibrocytes. A slow but steady loss of senescent cells could underlie the observed decrease of resident and grafted mFB in the cardiac scar. Outside the lesion area, no increased fibrosis was detected in the native myocardium either 2- or 8 weeks after mFB transplantation, as proven by quantitation of Sirus-red/Fast-green stainings of cardiac sections (Figure S6E,F). Next, we performed anti-Cx43 immunostainings in the cardiac scar of 8 week animals and found Cx43^+^/eGFP^+^ cells in LV-Cx43 and Cx43^-^/eGFP^+^ cells in LV-eGFP hearts (Figure 6C). Cx43 and eGFP protein content was akin to 2 weeks hearts quantified in excised scars using Western blotting and yielded a more than 3-fold increase in LV-Cx43 hearts compared to controls (Figure 6D).

*In vivo* electrophysiological testing evidenced a significantly lower VT incidence of 38.9% in LV-Cx43 mice, compared to 88.9% in CI (p-value = 0.0045) and 77.8% in LV-eGFP control mice (p-value = 0.041), respectively (Figure 6 E,F). We have also analysed the short- and long-term electrophysiological data obtained from the *in vivo* testing regarding VT duration (Figure S7B,D) and number of VT events (Figure S7A,C). Interestingly, VT events/animal were significantly lower in the LV-Cx43 group at both time points post-CI (1.45 events at 2 weeks; 1.17 events at 8 weeks) compared to the CI control group (4.29 events at 2 weeks, p-value = 0.04; 5.39 events at 8 weeks, p-value = 0.0017), consistent with improved electrical stability. In contrast, the duration of the individual self-terminating VT did not show statistically relevant differences between the groups (LV-Cx43: 0.69 s at 2 weeks, 1.3 s at 8 weeks; CI: 1.9 s at 2 weeks, 1.3 s at 8 weeks; LV-eGFP: 0.65 s at 2 weeks, 1.3 s at 8 weeks). Echocardiography revealed that in contrast to the 2-week stage, cardiac pump function was comparable in the different groups (Figure 6G,H,I), likewise infarct size (Figure S6C,D).

Thus, our data demonstrate that grafting of Cx43 expressing mFB into cardiac infarcts strongly reduces short- and long-term electrical vulnerability *in vivo,* while long-term cardiac pump function is similar between groups.

## Discussion

The cardiac scar has so far proven little amenable for targeting and/or gene therapy strategies. Herein, we show an efficient approach to target and modulate the functional properties of the scar by combining *ex vivo* gene therapy in mFB with *in vivo* cell therapy with the help of magnetic steering. FB make up a large number of cardiac cells and are known to play an important biological role in the heart 13. Many FB die upon ischemic stress, but surviving cells transdifferentiate into αSMA^+^ mFB, strongly proliferate, and repopulate the cardiac scar. Thus, cardiac mFB resist the adverse local conditions in ischemic lesions, stabilise these against rupture, play a key role in scar formation, and represent the dominant cell source in the persisting scar 12, 13. In contrast to CM, cardiac mFB express only a negligible amount of the coupling protein Cx43. This is one of the main reasons that electrical conduction is severely impaired in this area, favouring the occurrence of post-MI VT due to re-entry phenomena 33.

Given the distinct properties of cardiac cells and the futile efforts to effectively target the cardiac scar, we probed a combination of gene and cell therapy using cardiac mFB. We opted for embryonic mFB because of their strong proliferation rate, which enables *in vitro* expansion for grafting. In our earlier work, we demonstrated that magnetic steering using MNP-loaded cells strongly enhanced eCM engraftment rates 10. We have used PMAO-MNP as these particles have a good magnetic moment, low cellular toxicity, and, most importantly, do not form intracellular aggregates. Another advantage is that PMAO-MNP can be easily detected due to their attached fluorochrome and that these particles are degraded by lysozymes 18, 34.

Our initial experiments revealed unprecedented cell engraftment at 2 weeks after CI and injecting LV-treated 2.0x10^5^ mFB into the fresh lesion. Assuming that roughly half of the injected cells are eGFP^+^, grafted mFB numbers would be close to 100,000, far more than reported after injecting bone marrow-derived cells 35-37, mesenchymal stem cells 38, 39, or CM 10, 40. Our data suggest that both the tolerance of mFB towards the adverse conditions in cardiac lesions and their strong proliferation underlie these massive engraftment rates, as almost 50% of mFB were still proliferative after more than 2 weeks in cell culture, and 36% of engrafted eGFP^+^ mFB remained positive for Ki67 in the cardiac lesion. Moreover, the apoptosis rate was very low at different time points after transplantation. Our experiments also showed that the MNP/magnet approach enhanced mFB engraftment rates 3-fold, underscoring the impact of this technology. Because only 40% of grafted cells were eGFP labelled, we used a transgenic system to study the dynamics of exoFB engraftment and their interactions with endoFB. Analysis of exoFB at different time points of cell culture revealed that the *in vitro* enrichment yielded > 90% cardiac FB. Numbers of grafted cells were confirmed to be very high 1- and 2 weeks post-CI (~ 60,000 cells), but overall, somewhat lower than observed with mFB in preselected hearts using macroscopic inspection. The main difference between these two FB types is the 3-fold higher proliferation rate of mFB, indicating that mFB would be the cell type of choice for translational purposes. Using genetically labelled exoFB enabled us to explore their distribution pattern over time in the cardiac scar and their cell biological effects. ExoFB were positioned in the epicardial area of the lesion, which is probably due to the injection method, whereas endoFB were closer to the endocardial layer. Surprisingly, endo- and exoFB did not migrate and mix over time but remained in their separate locations. Importantly, grafting of exoFB enhanced the proliferation rate of endoFB by 4-fold, suggesting signalling between these two cell populations, while this procedure did not cause cardiac fibrosis outside of the scar. Even though the degree of engraftment of cardiac mFB short- and long-term after injection was far above the numbers reported for other cell types 41, 42 a steady loss of cells over time was observed. This was neither due to cell rejection because of prominent immune cell invasion nor apoptosis. Rather, the cardiac scar (CI) in the mouse heart is thinning out over time and becoming less cellular **(**1.5-fold thinner and 1.9-fold reduction of cells at 8 weeks compared to 2 weeks post-CI**)**. This is most likely due to the transdifferentiation of grafted and resident FB into senescent matrifibrocytes, which display a slow turnover and perish over time. In addition, the reduced vascularisation could also be a factor 43.

Given the prominent engraftment rates of mFB in the cardiac lesion, we interrogated, as proof of concept, whether a combination of *ex vivo* gene and *in vivo* cell therapy could even repair functional deficits of the infarcted heart. This is the case, as grafting Cx43 overexpressing mFB provided strong anti-VT protection *in vivo* even at the late stage, despite the lower number of grafted mFB. These results are consistent with our earlier work in which grafting of Cx43^+^ excitable cells 11 or direct LV-mediated transduction of resident endoFB 7 reduced post-infarct VT incidence. Our experimental evidence suggests that this anti-VT mechanism is due to increased conduction velocity in the border zone and the scar area, which prevents wave breaks and subsequent re-entry in the infarcted mouse heart 11. At this stage, we can only speculate about the potential cellular mechanisms, as it could be due to heterocellular coupling, a concept proposed in recent work 44-47, and/or electrotonic events. In any case, FRAP measurements prove that functional Cx43 gap junctions are formed in LV-Cx43 treated mFB and that these cells can couple. However, the small size of the mouse heart, as well as other technical issues with voltage mapping, have proven to be a limiting factor. Therefore, the mechanism underlying the Cx43-based anti-VT effect and its persistence over time would need to be explored in the future in larger animal models.

In summary, we present a novel approach that combines *ex vivo* gene therapy and *in vivo* cell therapy with mFB in combination with MNP/magnetic steering to efficiently target the cardiac scar and even modulate the function of the infarcted heart. An additional advantage of our strategy is that autologous cardiac mFB can be obtained through biopsy outgrowths or *in vitro* differentiation of patient-specific hiPSC. Besides anti-VT protection, one can envision various interesting therapeutic directions, such as modifying the immune response by overexpressing immunomodulating factors or targeting other cell biological and/or functional factors in mFB and grafting the cells. Such approaches would not require long-term engraftment and persistence of transplanted cells, as biological effects are warranted short-term after the lesion. Nevertheless, new strategies to keep engraftment rates more stable over time would be welcome.

## Conclusions

Cardiac myofibroblasts effectively engraft in cardiac lesions, continue to proliferate without affecting scar size, and induce enhanced proliferation in the endogenous myofibroblasts. Moreover, a combination of *ex vivo* gene- and *in vivo* cell therapy with Cx43 provides strong protection against post-infarct ventricular tachycardia at 2- and 8 weeks post-CI.

## Supplementary Material

Supplementary figures and tables.

## Abbreviations

AAV: adeno-associated virus
AV: atrio-ventricular
CM: cardiomyocytes
CI: cryoinjury
cTnT: cardiac troponinT
Cx43: Connexin 43
Δ AWT: Δ anterior wall thickening
eCM: embryonic cardiomyocytes
EF: ejection fraction
endoFB: endogenous fibroblasts
exoFB: exogenous fibroblasts
FB: fibroblasts
FS: fractional shortening
GO: gene ontology
Human induced-pluripotent stem cells: hiPSCs
Implantable cardioverter defibrillators: ICD
LDH: lactate dehydrogenase assay
LV: lentivirus
mFB: myofibroblasts
MI: myocardial infarction
MNP: magnetic nanoparticles
MOI: multiplicity of infection
MPS: magnetic particle spectrometry
MTT: multitox-Fluor multiplex cytotoxicity assay
PMAO: poly (maleic anhydride-alt-1-octadecene)
RT: room temperature
TEM: transmission electron microscopy
VT: ventricular tachycardia
