# Supplementary material for: Nanoparticle-assisted targeting of heart lesions with cardiac myofibroblasts: Combined gene and cell therapy
Source: Theranostics. 2025 Mar 18;15(10):4287–307. doi: 10.7150/thno.103816 (PMC11984384; doi:10.7150/thno.103816)
Supplement: Supplementary file 1 — Supplementary figures and tables. [file thnov15p4287s1.pdf]

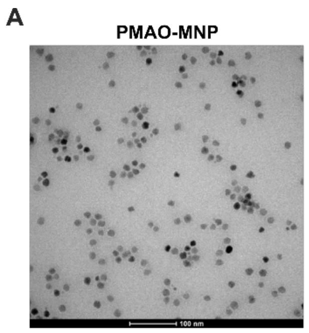

B

MNP	Inorganic core diameter (TEM, nm)	Hydrodynamic diameter (number, nm)	ζ potential (mV)
PMAO	12±1	23±1	-36
SoMag5	7±1	40±14	-38

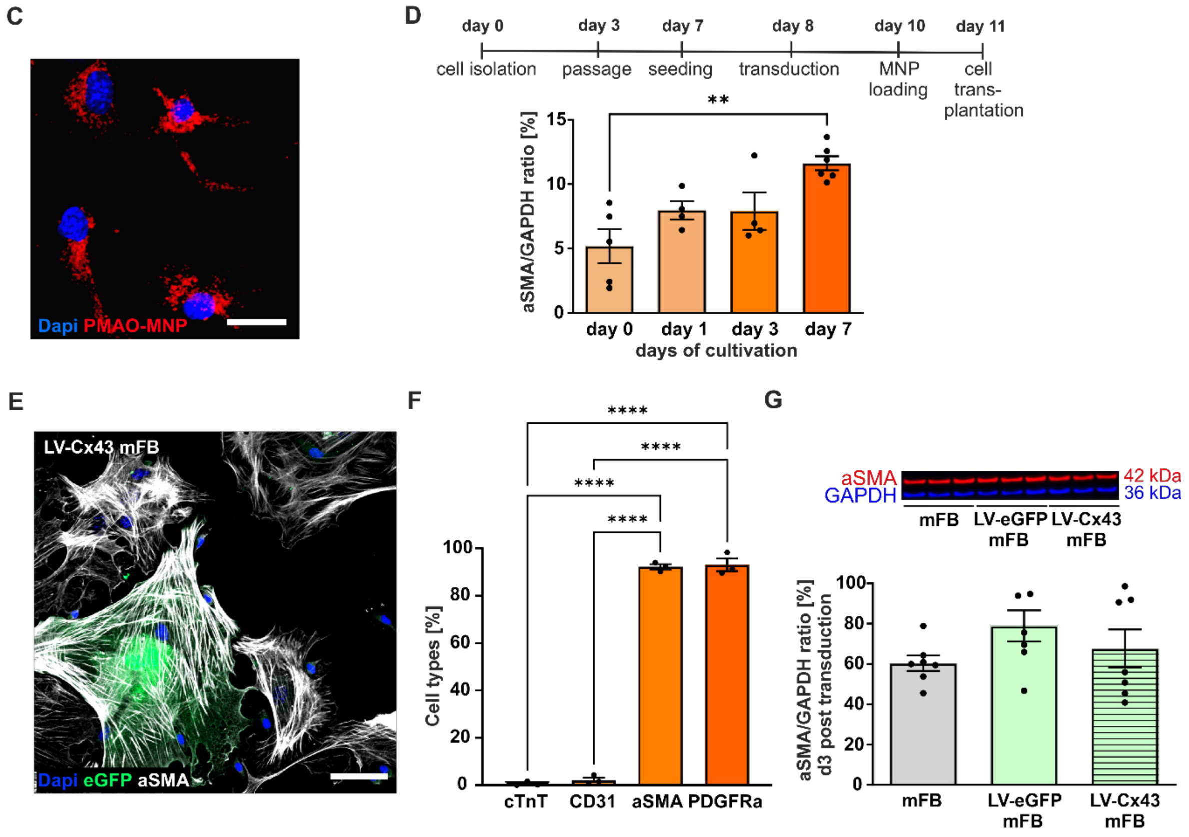

Figure S1. Cultivation, lentiviral (LV) transduction and magnetic nanoparticle (MNP)-loading of murine embryonic cardiac fibroblasts (mFB) *in vitro*. (A) Transmission electron microscopy (TEM) image of PMAO-MNP; (B) Physical characteristics of PMAO- and SoMag5-MNP. (C) mFB loaded with PMAO-MNP (25 pg Fe/cell, overnight) without magnetic field application (blue: nuclei, red: PMAO-MNP, bar: 20 µm). (D) Western Blot analysis of αSMA expression of mFB after different cultivation times; (GAPDH used as housekeeper). (E) Image of LV-Cx43 mFB 3 days post transduction (day 11; blue: nuclei, green: native eGFP fluorescence, white: αSMA, bar: 50 µm). (F) Enrichment of LV-Cx43 mFB (after MNP loading, day 10). Analysis at day 11 of cultivation with immunostainings against different cellular marker. (G) Analysis of αSMA protein expression of LV-treated and control mFB 3 days post transduction (day 11 of cultivation) using GAPDH as housekeeper. Data are presented as the mean ± SEM. P-value: ** = < 0.01; **** = 0.0001. Abbreviations: lentivirus (LV); magnetic nanoparticles (MNP); myofibroblasts (mFB); Transmission electron microscopy (TEM).

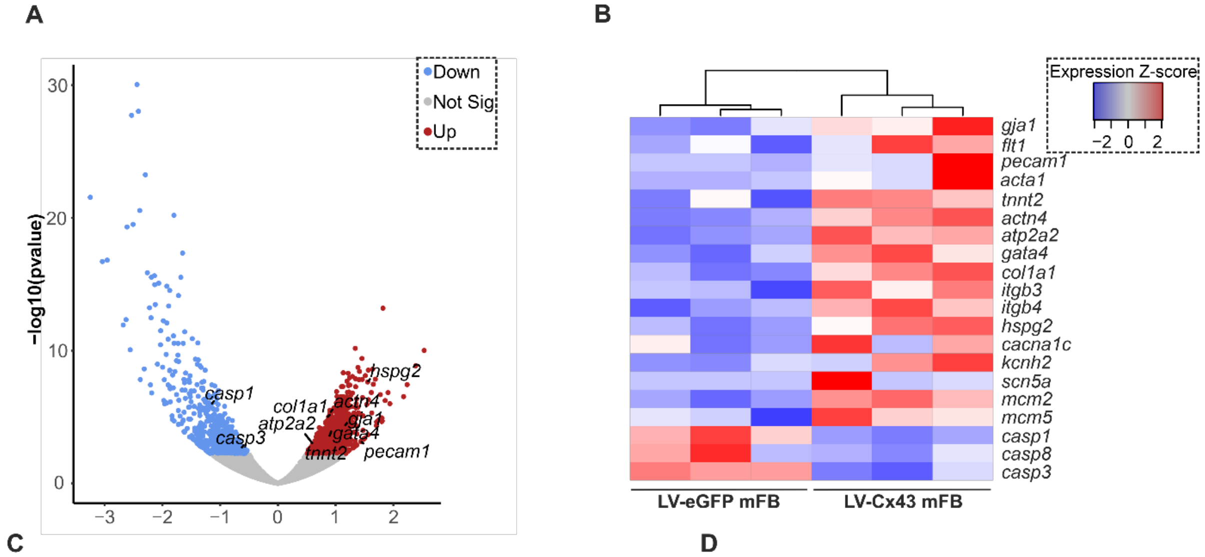

C

GO (LV-Cx43 mFB downregulated genes)	#Genes
Regulation of transcription from RNA polymerase II promoter	89
Immune system process	78
Innate immune response	75
Defense response to virus	52
Inflammatory response	37
Apoptotic process	33
Activation of innate immune response	19

D

GO (LV-Cx43 mFB upregulated genes)	#Genes
Cell differentiation	58
Angiogenesis	45
Cell migration	43
Heart development	37
Extracellular matrix organization	21
Wound healing	14
Collagen fibril organization	11

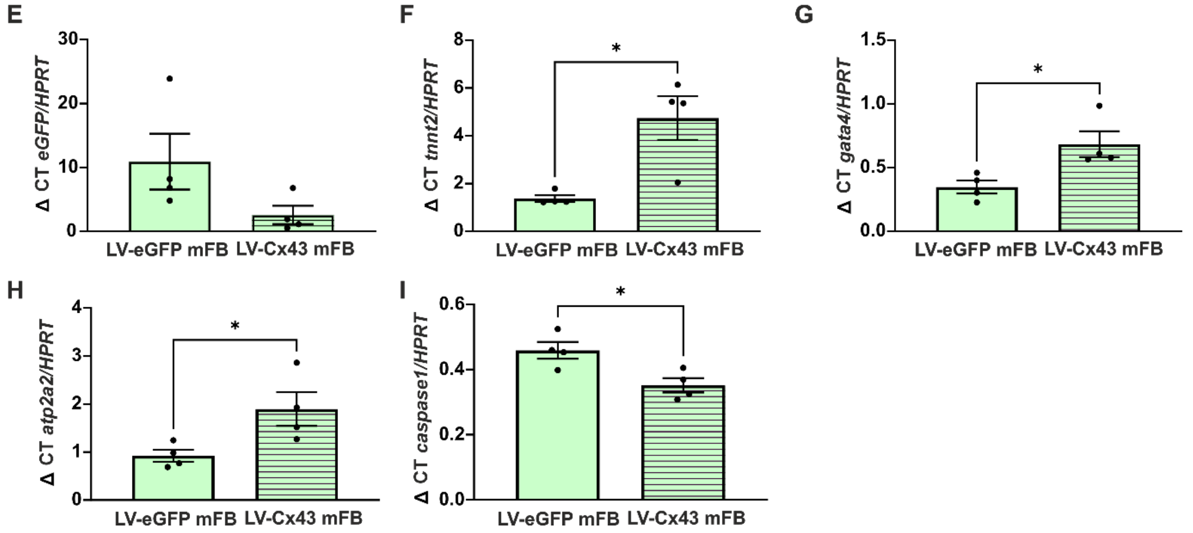

Figure S2. Characterisation of mFB using RNAseq 3 days post LV transduction. (A) Volcano plot and (B) heatmap of up- and downregulated genes of LV-Cx43 *vs* LV-eGFP mFB (both n = 3). Gene ontologies (GOs) of down (C)- and upregulated (D) genes in LV-Cx43 mFB. (E) RT-qPCR analysis to detect and quantify *eGFP*, (F) *tnnt2*, (G) *gata4*, (H) *atp2a2* and (I) *caspase1* RNA expression. Graphs show ΔCT values in relation to the housekeeping gene *HPRT*. Data are presented as the mean ± SEM. P-value: * = < 0.05. Abbreviations: connexin43 (Cx43); gene ontologies (GOs); lentivirus (LV); myofibroblasts (mFB).

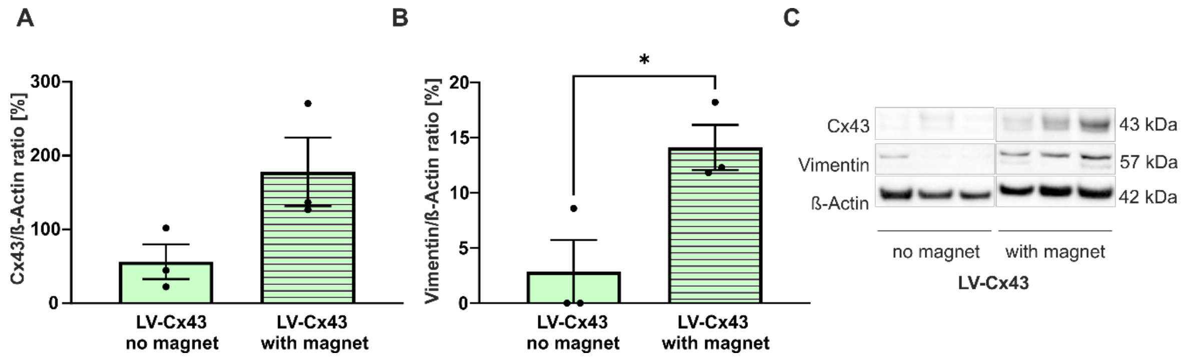

Figure S3. Enhanced engraftment 2 weeks after no- or magnet-assisted injection of LV-Cx43 mFB. Quantitation of (A) Cx43 and (B) Vimentin protein content in excised scar tissue of LV-Cx43 injected hearts assessed by Western Blotting using beta-Actin as housekeeper; (C) Original Western Blot. Data are presented as the mean ± SEM. P-value: * = < 0.05. Abbreviations: connexin43 (Cx43); lentivirus (LV); myofibroblasts (mFB).

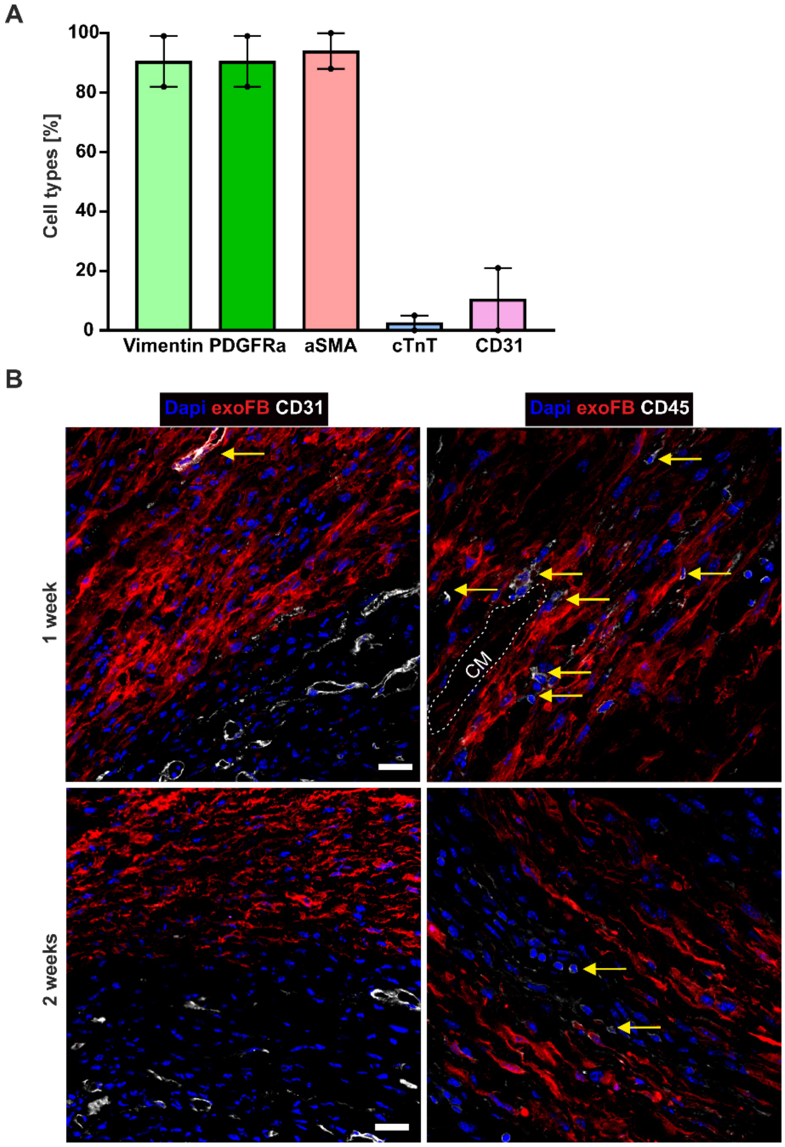

Figure S4. Characterisation of tamoxifen induced transgenic exoFB in cell culture and in the myocardial scar 1- and 2 weeks post-CI. (A) Assessment of enrichment of eGFP$^+$/tomato$^+$ exoFB using immunostainings against different cell markers. Cells were obtained/isolated from tamoxifen induced transgenic mTmGxTcf21^{MCM} hearts (n = 4) and cultured for 5 days. (B) Few tomato$^+$/CD31$^+$ endothelial and tomato$^-$/CD45$^+$ immune cells (upper panel, marked by arrows) are/were detected 1 week, no tomato$^+$/CD31$^+$ and very few tomato$^-$/CD45$^+$ cells 2 weeks (lower panel, marked by arrows) after CI and intramyocardial cell injection (blue = nuclei, red = native tomato fluorescence, white = CD31 or CD45). Bars: 20 µm. Data are presented as the mean ± SEM. Abbreviations: cryoinjury (CI); neonatal cardiac myofibroblasts (exoFB).

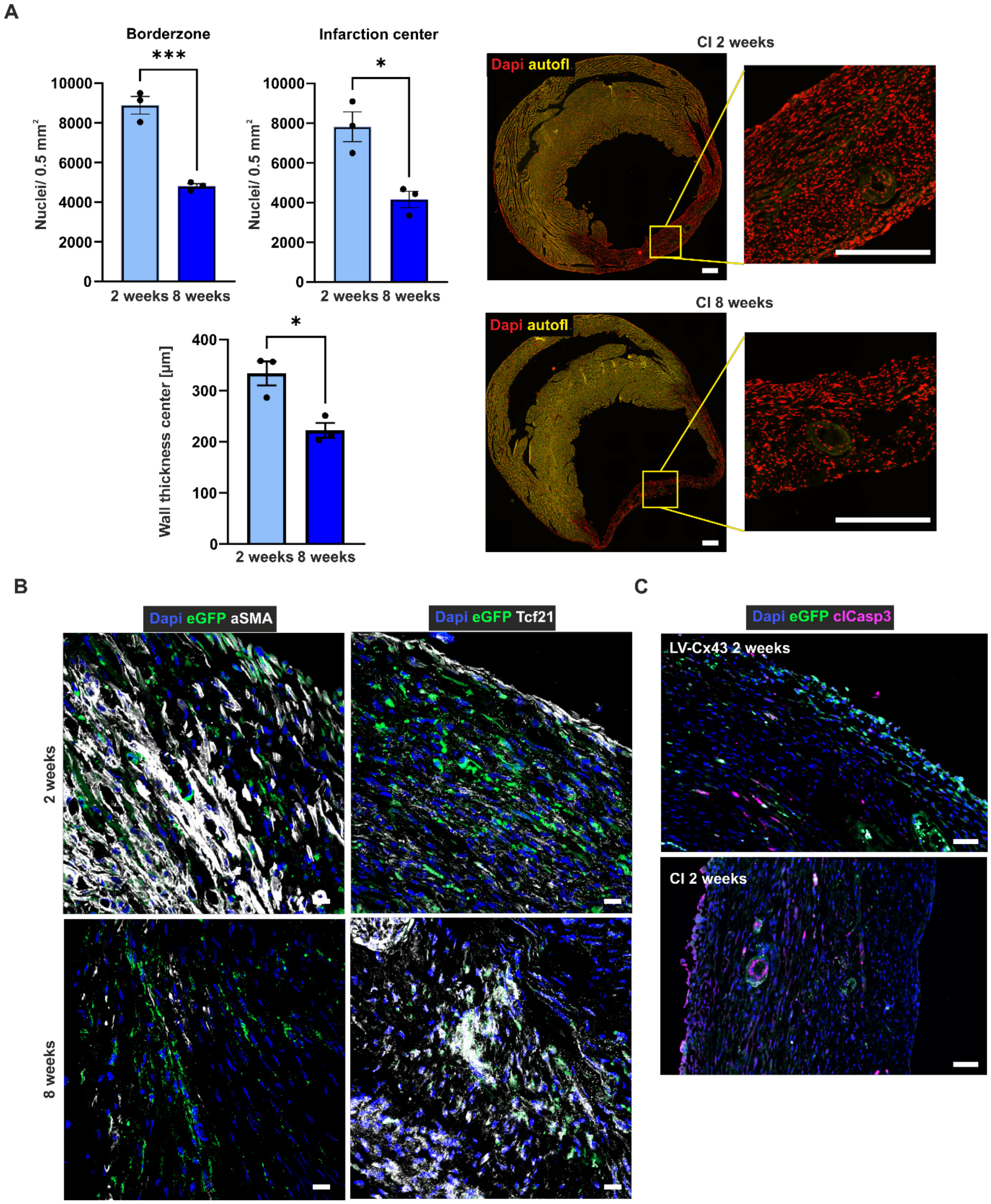

Figure S5. Cellularity of the myocardial scar, characterisation of transplanted mFB over time, apoptosis assessment. (A) Wall thickness of the centre, and cellularity (Dapi staining) in the border zone and the centre of the cardiac scar 2- and 8 weeks post-CI (red = nuclei, yellow = autofl., bars = 500 µm). (B) αSMA (left) and Tcf21 (right) expression of engrafted mFB 2- and 8 weeks post-injection (blue = nuclei, green = eGFP, white = αSMA/Tcf21, bars = 20 µm). (C) Caspase 3 staining of sections of CI and LV-Cx43 mFB injected hearts 2 weeks post-CI (blue = nuclei, green = eGFP staining, violet = cleaved Caspase 3 staining, bars = 50 µm). Data are presented as the mean ± SEM. P-values: * = < 0.05; *** = < 0.001. Abbreviations: cryoinjury (CI); connexin43 (Cx43); lentivirus (LV); myofibroblasts (mFB).

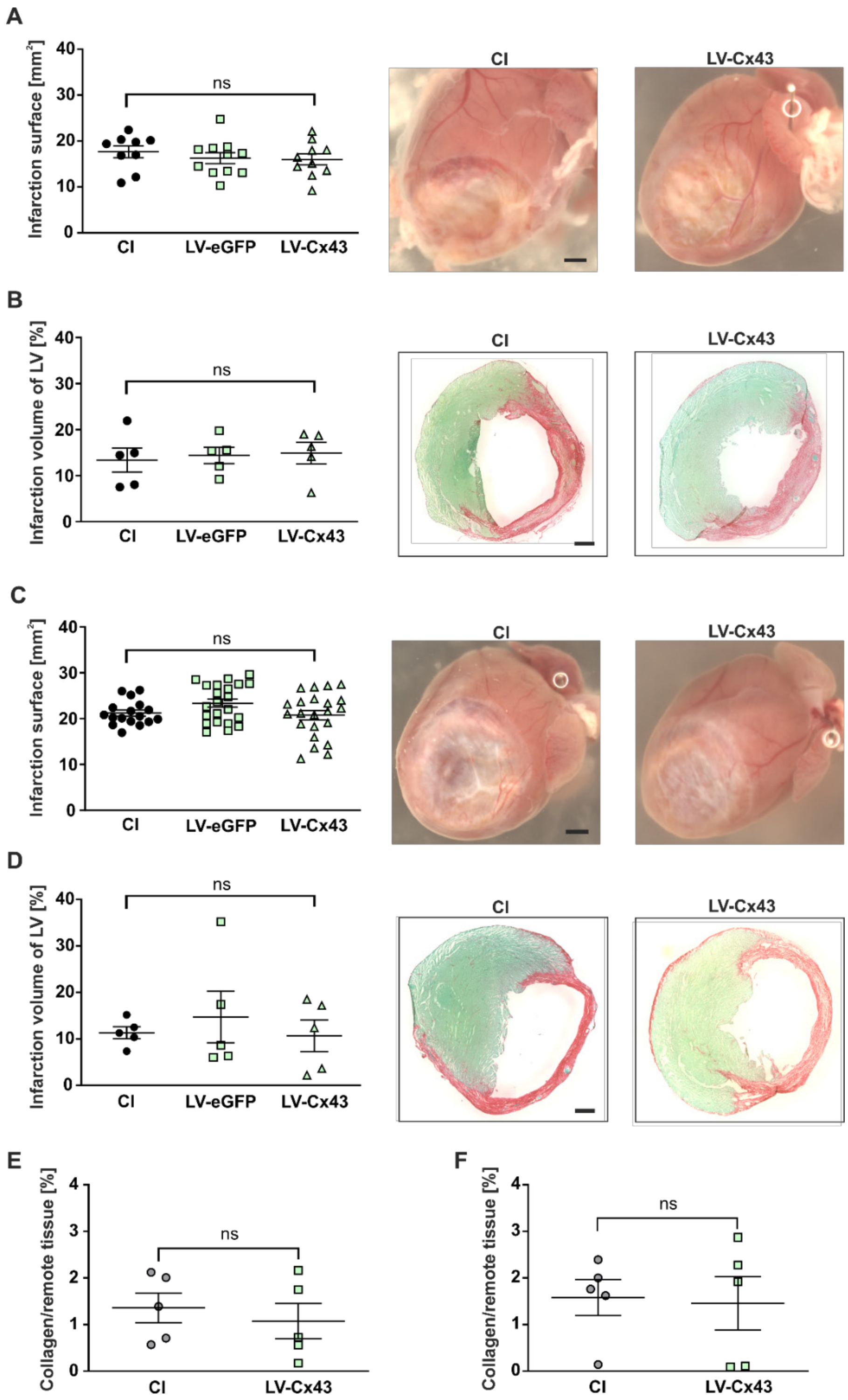

Figure S6. Characterisation of the myocardial scar (CI) and remote areas 2- and 8 weeks after injury without and with injection of LV-eGFP or LV-Cx43 mFB. (A) Analysis of infarction surface and (B) infarction volume 2 weeks post-CI and post-injection of cells. (C) Analysis of infarction surface and (D) infarction volume 8 weeks post-CI and post-injection of cells. (E, F) Quantitative assessment of collagen content in remote areas (green areas of pictures in B,D) of (E) 2- and (F) 8 weeks of CI control and LV-Cx43 mFB injected hearts using imaging analysis. A, C: bars: 1000 µm; B, D, E, F: bars : 500 µm. Data are presented as the mean ± SEM. Abbreviations: cryoinjury (CI); connexin43 (Cx43); lentivirus (LV); myofibroblasts (mFB).

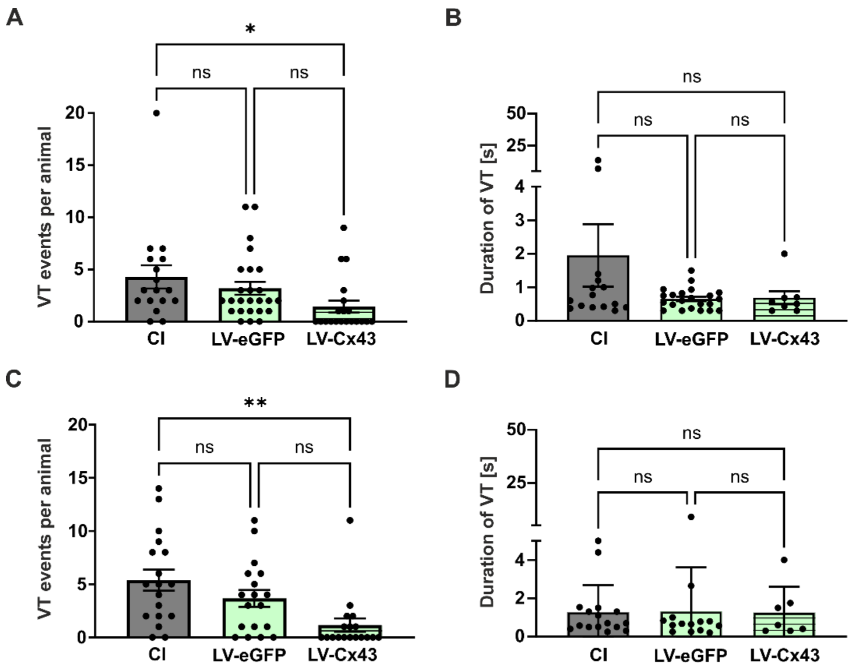

Figure S7. Analysis of VT events/animal and VT duration 2- and 8 weeks post-CI and injections of LV-eGFP and LV-Cx43 cells: Induced VT events/animal at 2 (A) – and 8 (C) weeks, and mean VT duration at 2 (B) – and 8 (D) weeks post-CI obtained with electrophysiological *in vivo* testing. Data are presented as the mean ± SEM. P-values: * = < 0.05; ** = < 0.01. Abbreviations: cryoinjury (CI); connexin43 (Cx43); lentivirus (LV); ventricular tachycardia (VT).
